# Structure–Reactivity Relationships of Oxime–Oxalate/Glyoxylate/Ester Derivatives: Dual Photo/Thermal Initiators for Visible Light Polymerization and Composite Preparation

**DOI:** 10.1002/anie.202521296

**Published:** 2025-11-30

**Authors:** Tong Gao, Thybault De Monfreid, Ji Feng, Jing Zhang, Fabrice Morlet‐Savary, Céline Dietlin, Michael Schmitt, Frédéric Dumur, Jean‐Patrick Joly, Malek Nechab, Pu Xiao, Jacques Lalevée

**Affiliations:** ^1^ State Key Laboratory of High Performance Ceramics Shanghai Institute of Ceramics Chinese Academy of Sciences Shanghai 200050 P.R. China; ^2^ Université de Haute‐Alsace CNRS IS2M UMR7361 Mulhouse F‐68100 France; ^3^ Université de Strasbourg Strasbourg France; ^4^ Aix Marseille Univ CNRS ICR UMR 7273 Marseille F‐13397 France; ^5^ Future Industries Institute University of South Australia Mawson Lakes South Australia 5095 Australia

**Keywords:** 3D Printing, dual photo/thermal initiator, low‐cytotoxic, photopolymerization, sunlight

## Abstract

In this study, we reported the design, synthesis, and comprehensive evaluation of a series of nitro carbazole‐based oxime photoinitiators (OPIs, OP1: oxime oxalate, OP2: oxime glyoxylate), as a series of efficient Type I photoinitiators (PIs) for the free radical photopolymerization (FRP) of trimethylolpropane triacrylate (TMPTA) and ethoxylated trimethylolpropane triacrylate (ETPTA) under blue light‐emitting diodes and sunlight irradiation. Computational molecular modeling was employed to predict the effect of OPIs structures on the photoinitiation properties. The predictions suggest that OP1 had a higher propensity for decarboxylation and therefore a better photoinitiation behavior. Compared to OP2, OP3, and commercial benchmark photoinitiator TPO, OP1 exhibits exceptional photoinitiation performance when exposed to LED@405 nm, LED@450 nm, and sunlight. OP1 undergoes decarboxylation to efficiently produce CO_2_ and free radicals, thereby initiating the photopolymerization reaction. Remarkably, OP1 is successfully applied in 3D printing, producing complete morphology with high‐resolution structure, showcasing its potential for advanced manufacturing applications. The photochemical mechanism of OPIs is comprehensively elucidated using the monitoring of the CO_2_, steady state photolysis, UV–vis absorption spectroscopy, fluorescence spectroscopy, and electron spin resonance techniques. These experimental investigations are supported by data OP1 from the molecular modelling carried out. Additionally, thermal polymerization shows that OP1 had a high thermal initiation capability, and the composites are successfully prepared together with carbon fibers. The cytotoxicity of the synthesized oxime oxalate and TPO on human umbilical vein endothelial cells (HUVECs) results in a lower cytotoxicity for the oxalate than for TPO. Therefore, OP1, which has never been reported before, can be used as a highly efficient and low cytotoxic dual photo/thermal initiator. This research not only provides theoretical and practical insights into the design and development of new efficient Type I PIs, but also opens up new perspectives for curing applications that require scalability, cost‐effectiveness, environmental sustainability, and green chemistry.

## Graphical abstract

OP1, a newly synthesized nitro‐carbazole oxime oxalate, shows superior Type I photoinitiation under blue LEDs and sunlight due to efficient decarboxylation. It enables high‐resolution 3D printing, offers dual photo/thermal initiation, lower cytotoxicity than TPO, and provides insights for designing sustainable photoinitiators.

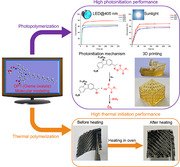

## Introduction

Photopolymerization refers to a technique utilizing photoinitiators (PIs) to initiate a polymerization reaction of monomers under light radiation.^[^ ^1^, ^2^ ^]^ This technique has drawn attention due to its fast reaction speed, spatial controllability, and energy efficiency,^[^ ^3^, ^4^ ^]^ and is used in a variety of applications, such as 3D printing,^[^ ^5^, ^6^, ^7^, ^8^, ^9^, ^10^, ^11^ ^]^ metamaterial,^[^ ^12^ ^]^ biomedical engineering,^[^ ^13^, ^14^ ^]^ holography,^[^ ^15^, ^16^ ^]^ microfabrication,^[^ ^17^ ^]^ coatings/inks,^[^ ^18^, ^19^ ^]^ and adhesives.^[^ ^20^ ^]^ Compared to traditional photopolymerization using ultraviolet (UV) lamps as a light source, photopolymerization using light‐emitting diodes (LED) visible light as an artificial light source has the advantages of being safer, more environmentally friendly, more process flexibility, and low equipment costs.^[^ ^21^, ^22^, ^23^ ^]^ Moreover, the LED light sources provide stable spectral output, which reduces the impact of light intensity fluctuations on the polymerization reactions and enables a precise control of wavelengths, and they are thus suitable for different types of photoinitiators.^[^ ^24^, ^25^ ^]^ LED visible light photopolymerization is increasingly taking on an increasingly important role. Moving one step further, it is possible for sunlight to be employed as a low‐cost and limitless source of light to carry out the polymerization process.^[^ ^26^ ^]^ Compared to artificial light sources, sunlight offers several advantages, including low cost, a broad emission spectrum, and energy efficiency, making it an ideal candidate for photopolymerization.^[^ ^27^, ^28^ ^]^ Markedly, sunlight, as a full‐spectrum light source, activates various photoinitiators, enabling a large‐scale production, efficient curing, and outdoor applications in sunlight‐abundant environments.^[^ ^29^, ^30^ ^]^

Photoinitiators act as a very important part of the photopolymerization process as these structures absorb the light energy and produce active species to initiate the polymerization reaction.^[^ ^31^, ^32^ ^]^ In free radical photopolymerization (FRP), two different photoinitiators can be used to generate free radicals, i.e., Type I PIs followed by α‐cleavage with radical formation capability and Type II PIs followed by H‐abstraction reaction.^[^ ^33^, ^34^ ^]^ However, Type II PIs typically need to be combined with coinitiators to form multicomponent photoinitiating systems (PISs) and additionally can suffer from hydrogen donors, low reactivity, and electron and/or proton transfer efficiency, which not only limit the use of PIs in such complex formulations, but also lead to relatively low reaction performance.^[^ ^35^, ^36^, ^37^ ^]^ Therefore, highly reactive, one‐component Type I PIs with excellent initiation performance and suitability for visible light‐induced FRP have become a popular research focus.

Oxime ester compounds, as typical Type I photoinitiators, have been widely explored for free radical photopolymerization owing to their high photoactivity.^[^ ^38^, ^39^ ^]^ Besides, the carbon dioxide produced after the decarboxylation reaction could serve to eliminate the oxygen inhibition effect for free radical polymerization.^[^ ^40^ ^]^ However, the commercial oxime ester‐based photoinitiators OXE‐01 and OXE‐02 exhibit absorption bands predominantly in the ultraviolet range (<350 nm), with limited overlap in the near‐UV or visible spectral range.^[^ ^41^, ^42^, ^43^ ^]^ This spectral mismatch results in significantly reduced photoinitiation efficiency under long‐wavelength LED sources commonly employed in visible light‐induced free‐radical photopolymerization. To address this limitation, molecular modeling strategies have been proposed to incorporate chromophores into the oxime ester framework, thereby extending their absorption toward the visible region to better match the emission wavelength of modern LED devices.^[^ ^44^ ^]^ Nevertheless, such structural modifications are not trivial, as they must simultaneously ensure efficient free radical generation, photoinitiation performance, and low cytotoxicity. The cytotoxicity of photoinitiators, in particular, poses a significant constraint for their use in biomedical applications.^[^ ^45^ ^]^ Therefore, the development of novel Type I photoinitiators that synergistically combine strong visible light absorption, high initiation efficiency, and excellent biocompatibility under long‐wavelength LED or even sunlight irradiation remains both a scientific challenge and a technological imperative.

**Scheme 1:**
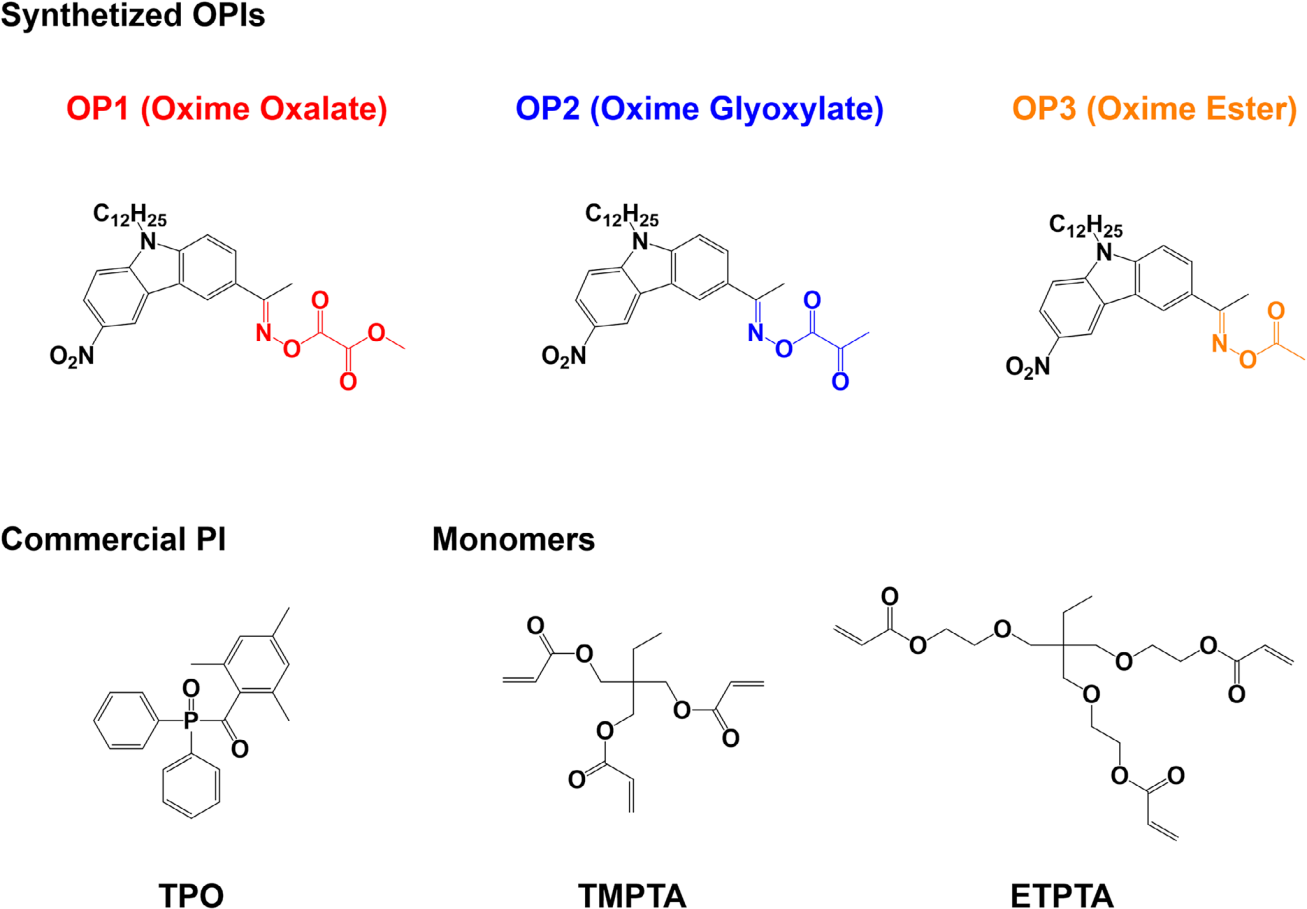
Chemical structures of oxime oxalate (OP1), oxime glyoxylate (OP2), oxime ester (OP3),^[^ ^46^ ^]^ commercial photoinitiator TPO, and monomers (TMPTA and ETPTA) used in this study.

In this study, three nitro carbazole‐based oxime photoinitiators (OPIs) (See Scheme 1), oxime oxalate (OP1), oxime glyoxylate(OP2), and oxime ester (OP3) (OP3 has been reported as a Type I photoinitiator in our previous study),^[^ ^46^ ^]^ were prepared as Type I PIs by introducing oxime oxalate and glyoxylate groups as new substituents in the carbazole‐based oxime compound. The structures of OPIs were further investigated for their effects on the associated photoactivity. Molecular modeling calculations was carried out to predict the photoinitiation properties of OPIs by the frontier molecular orbitals, decarboxylation exothermicity, and cleavage exothermy. Photopolymerization kinetics of OPIs/TMPTA and OPIs/ETPTA under LED@405 nm, LED@450 nm, and sunlight were investigated in comparison with commercial Type I PI 2,4,6‐trimethyl(phenyl) diphenyl oxide (TPO). Subsequently, the 3D printing experiments of OPIs/TMPTA were performed. Then, the photochemical mechanism of OPIs compounds was thoroughly investigated and clarified by a combination of monitoring the decarboxylation reaction, steady state photolysis, UV–vis absorption spectroscopy, fluorescence spectroscopy, and electron spin resonance (ESR). Furthermore, the thermal initiation properties of OPIs/TMPTA were investigated by thermal polymerization experiments. Finally, the cytotoxicity of OPIs with and without light‐treated in human umbilical vein endothelial cells (HUVECs) was evaluated by Cell Counting Kit‐8 (CCK‐8) assay. For comparison, our previous work focused on glyoxylate oxime ester photoinitiators,^[^ ^7^ ^]^ whereas the present study introduces oxime oxalate as a fundamentally distinct class with a single‐step decarboxylation pathway and dual photo/thermal initiation ability.

## Results and Discussion

### Synthesis of OPIs

**Scheme 2:**
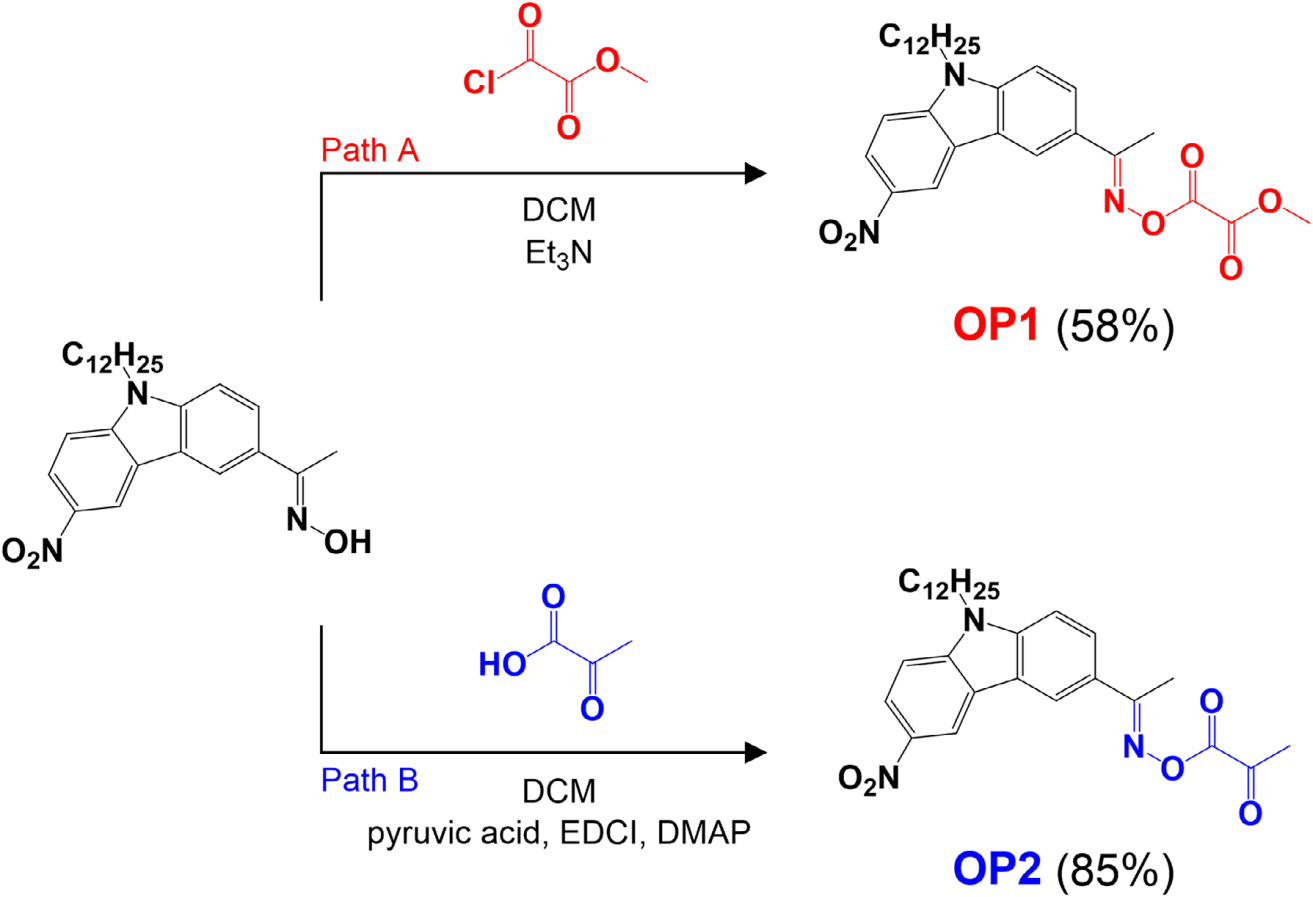
Synthesis protocol of OP1 and OP2.

The synthesis route of oxime oxalate (OP1) and oxime glyoxylate (OP2) is depicted in Scheme 2. OP1 was prepared following path A, starting from the previously reported 1‐(9‐dodecyl‐6‐nitro‐9*H*‐carbazol‐3‐yl)ethan‐1‐one oxime^[^ ^46^ ^]^ and by adding triethylamine (Et_3_N) and methyl chlorooxyacetate in dichloromethane (DCM). OP2 was synthesized using 1‐(9‐dodecyl‐6‐nitro‐9*H*‐carbazol‐3‐yl)ethan‐1‐one oxime, pyruvic acid, 1‐ethyl‐3‐(3‐dimethylaminopropyl)carbodiimide (EDCI), and 4‐dimethylaminopyridine (DMAP) coupling, path B. Supporting information provides synthesis details and characterizations.

### Molecular Modeling and Cleavage Enthalpy

**Figure 1:**
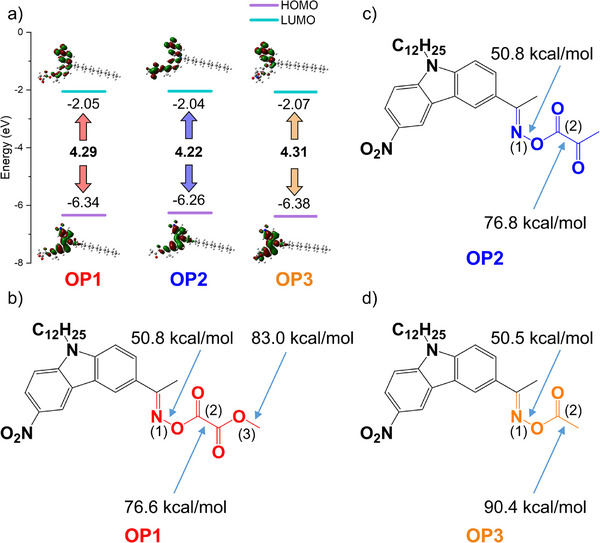
a) The HOMOs, LUMOs, and energy gaps of OPIs. Optimization of the geometry at a B3LYP/6‐31G* level of theory then single point at a MPW1PW91/6‐31g(d) level of theory; isovalue = 0.02 for the orbitals drawing. Bond dissociation energies of b) OP1, c) OP2, and d) OP3. Positions (1), (2), and (3) correspond to the N─O, C─C, and C─O bonds at a B3LYP/6‐31G* level of theory.

Figure 1a shows that the highest occupied molecular orbitals (HOMO) and lowest unoccupied molecular orbitals (LUMO) of OPIs have the typical π and π* orbitals, leading to a π–π* transition with a small intramolecular charge transfer (ICT) transition. The HOMO electron density of all OPIs are mainly localized on the carbazole moiety. The LUMO electron density of OP1 and OP3 are mainly localized on the nitro and carbazole groups. The carbazole moiety acted as an electron donor while the nitro group or the oxime glyoxylate moiety could act as electron acceptors. The LUMO electron density of OP2 were concentrated not only on the nitro and the carbazole moieties, but also on the oxime glyoxylate group with a large electron density. Even though the HOMO–LUMO energy gaps of OPIs were all close to that of OXE02 (4.20 eV), the ICT characterized by π–π* interactions in OPIs compared to the molecular orbitals of OXE02 still suggested that the maximum absorption wavelength of OPIs might be red‐shifted.^[^ ^47^ ^]^ Additionally, the energy gap of OP2 was lower than that of OP1 and OP3, which leads to a maximum absorption wavelength of OP2 larger than that of OP1 and OP3. Interestingly, the push‐pull effect evidenced in Figure 1a leads to the ICT transition band characterized by π–π*. It may be shown with an enhanced intensity of the absorption band of OPI3 in the UV–vis absorption spectra of OPIs.

**Figure 2:**
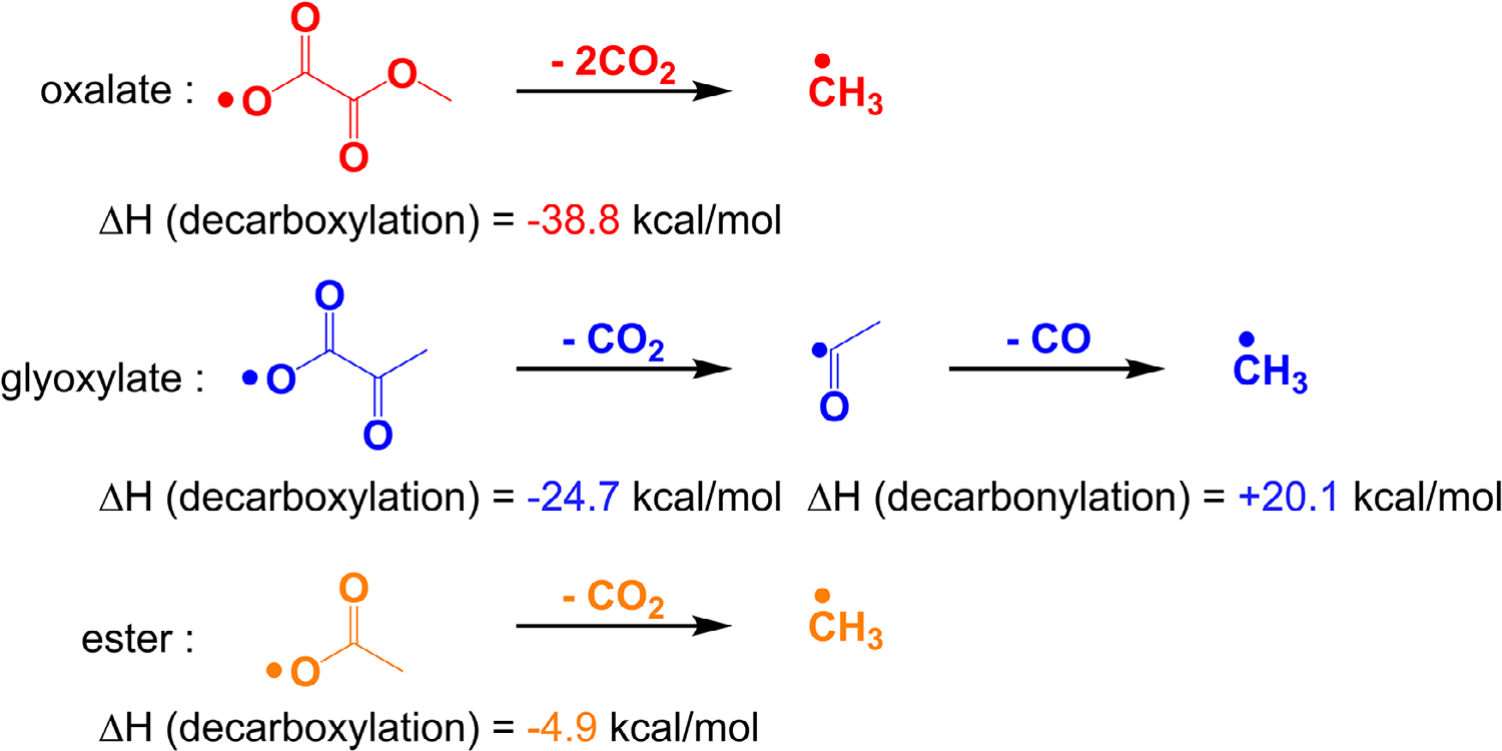
The enthalpy of decarboxylation reactions of the oxalate, glyoxylate, and ester chromophores.

As shown in Figure 1b–d, the bond dissociation energies (BDE) of the N─O bonds of all OPIs were much lower than those of the C─C and C─O bonds, suggesting that the N─O bond was more prone to cleave. An important parameter for assessing the initiation capability of Type I photoinitiators is the cleavage enthalpy (Δ*H*_cleavage T1_ or Δ*H*_cleavage S1_ = BDE (N─O or C─C or C─O) – E_T1_ or E_S1_, where Δ*H*_cleavage T1_ and Δ*H*_cleavage S1_ indicate the cleavage enthalpy of triplet state (T1) and singlet state (S1) respectively, and E_T1_ and E_S1_ denote the triplet state energy and singlet state energy respectively). Normally, a smaller Δ*H*_cleavage T1_ value corresponds to a better photoinitiation behavior of the photoinitiator.^[^ ^48^, ^49^ ^]^ According to Table 1, it can be inferred that the initiation performance of OP1 (N─O bond: Δ*H*_cleavage T1_ = ‐7.3 kcal mol)^−1^and OP3 (N─O bond: Δ*H*_cleavage T1_ = ‐7.7 kcal mol^−1^) may be superior to that of OP2 (N─O bond: Δ*H*_cleavage T1_ = ‐2.2 kcal mol^−1^). Combined with Figure 2, the enthalpy of decarboxylation reaction of oxalate was lower than that of ester, indicating that the reactivity of OP1 may be better than that of OP3. When Δ*H*_cleavage T1_ or Δ*H*_cleavage S1_ values were positive (C─C and C─O cleavage for S1 and T1, Δ*H*_cleavage T1_ or Δ*H*_cleavage S1_ > 0), cleavage reactions may still occur if enthalpy is not the exclusive criterion for cleavage reactions to proceed, especially if decarboxylation facilitates the reaction.^[^ ^35^ ^]^ In summary, it can be seen that the initiation properties of OP1, OP3, and OP2 gradually weakened. Moreover, all OPIs could possibly generate carbon dioxide (CO_2_) by decarboxylation reaction, but it would be very difficult to produce carbon monoxide (CO) for OP2.

**Table 1:** Bond Dissociation Energies of N─O, C─C, and C─O bonds, *E*_T1_ and Δ*H*_cleavage T1_ of OPIs (N─O, C─C, and C─O bonds) computed at a B3LYP/6‐31G* level of theory.

OPIs	*E*_T1_ (kcal mol^−1^)	N─O BDE (kcal mol^−1^)	N─O Δ*H*_cleavage T1_ (kcal mol^−1^)	C─C BDE (kcal mol^−1^)	C─C Δ*H*_cleavage T1_ (kcal mol^−1^)	C─O BDE (kcal mol^−1^)	C─O Δ*H*_cleavage T1_ (kcal mol^−1^)
OP1	58.1	50.8	−7.3	76.6	18.5	83.0	24.9
OP2	53.0	50.8	−2.2	76.8	23.8	n.d.	n.d.
OP3	58.2	50.5	−7.7	90.4	n.d.	90.4	32.2

n.d.: not determined.

**Figure 3:**
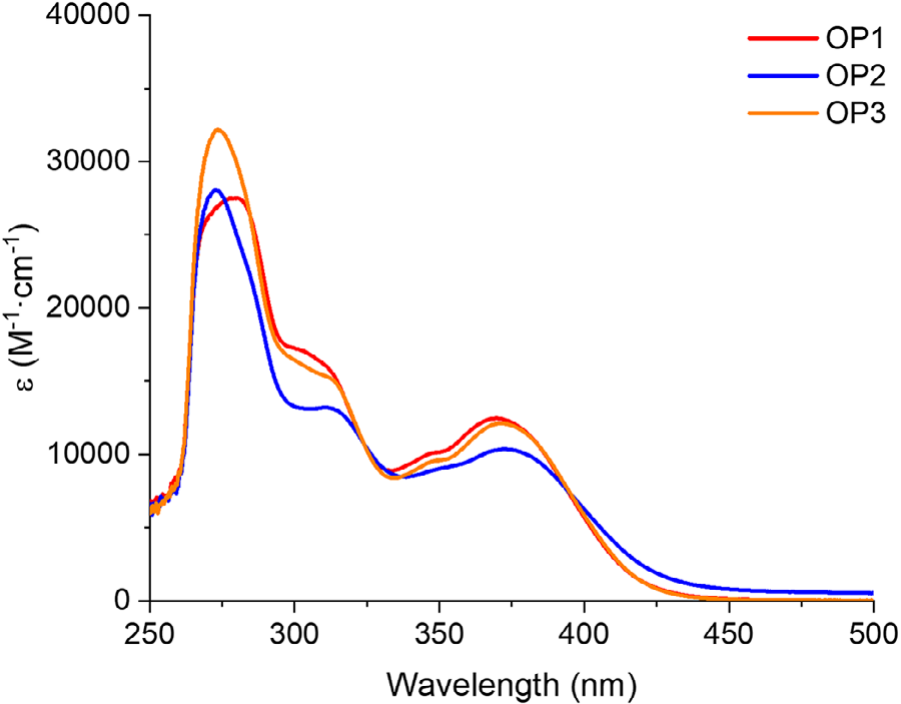
UV–vis absorption spectra of OPIs in ACN (concentration = 5 × 10^−5^ M).

The single state energy (E_S1_) of OPIs were determined using normalization of the crosspoint of the UV–vis absorption spectrum and the fluorescence spectrum (See Figures 3 and S1). The N─O bond (Δ*H*_cleavage S1_ < 0) in OPIs of the singlet state was more prone to be cleaved compared to those of the C─C and C─O bonds (Δ*H*_cleavage S1_ > 0) (See Table 2). Despite the positive enthalpy value of cleavage in the singlet state (C─C and C─O cleavage for S1, Δ*H*_cleavage S1_ > 0), the cleavage reaction may be possible if the decarboxylation is favorable for the reaction to occur. Moreover, it was observed that the chemical bonds (N─O, C─C, and C─O bonds) of OPIs in the singlet state could be cleaved more easily than those of OPIs in the triplet state from the energy point of view. Additionally, the fluorescence lifetime of OP1 was less than the instrumental response time (1.4 ns) (See Table S1). Such short fluorescence lifetime may be primarily attributed to intramolecular charge transfer,^[^ ^50^, ^51^ ^]^ which suggested fast and efficient cleavage of the excited state, corresponding to the better cleavage of S1.

**Table 2:** Δ*H*_cleavage S1_ of OPIs (N─O, C─C, and C─O bonds).

OPIs	N─O Δ*H*_cleavage S1_ (kcal mol^−1^)	C─C Δ*H*_cleavage S1_ (kcal mol^−1^)	C─O Δ*H*_cleavage S1_ (kcal mol^−1^)
OP1	−15.1	10.7	17.1
OP2	−15.8	10.2	n.d.
OP3	−14.8	n.d.	25.1

n.d.: not determined.

### Photophysical Properties

Figure 3 shows the UV–vis absorption spectra of OPIs in acetonitrile (ACN) and Table 3 lists the specific photophysical data of OPIs at the maximum absorption wavelength (*λ*_max_), 405 nm (*λ*_405_), and 450 nm (*λ*_450_). In previous studies, Lin et al. synthesized oxime oxalate compounds containing aryl groups (e.g., methyl 2‐(((diphenylmethylene)amino)oxy)‐2‐oxoacetate and 1‐(((diphenylmethylene)amino)oxy)‐2‐phenylethane‐1,2‐dione).^[^ ^52^ ^]^ Utilizing the organic photocatalyst thioxanthone with high triplet energy as a photosensitizer, the oxime oxalate compounds reacted with thioxanthone via triplet‐triplet energy transfer to synthesize 1,2‐carboxamidation products. Unlike this photosensitizer‐dependent energy transfer mechanism, the Type I photoinitiator in this study underwent direct Type I cleavage upon visible light irradiation. However, previously reported compounds lack absorption capacity in the blue light region (*λ*_max_ < 350 nm). Therefore, this study introduced a carbazole group with a large conjugated structure into the oxime oxalate. Compared to the *λ*_max_ of OXE‐02, which was less than 350 nm, the *λ*_max_ of OP1, OP2, and OP3 were red‐shifted to 370, 373, and 371 nm, respectively. It could be attributed to the introduction of nitro electron‐withdrawing substituent in OPIs, which increased the intramolecular charge transfer and thus red‐shifted the *λ*_max_ of the OPIs. As revealed in Figure 3 and Table 3, the *λ*_max_ of OP2 was slightly longer than that of OP1 and OP3. Additionally, it can be observed that the intensity of the absorption band of OP3 in the UV region was higher than that of OP1 and OP2. These results were all in high agreement with those predicted by molecular modeling. As shown in Table 3, all OPIs exhibited high molar extinction coefficients (*ε*) at the maximum absorption wavelength (*ε*_max_) and at 405 nm (*ε*_405_). Moreover, all OPIs showed molar extinction coefficients at 450 nm (*ε*_405_). The above results suggested that OPIs could be considered as appropriate photoinitiators for photopolymerization experiments exposed to LED@405 nm and LED@450 nm, and the maximum absorption wavelengths of OPIs were red‐shifted due to the electronic effect of the electron‐withdrawing substituent.

**Table 3:** Photophysical properties of OPIs in ACN (concentration = 5 × 10^−5^ M).

OPIs	*λ*_max_ (nm)	*ε*_max_ (M^−1^·cm^−1^)	*ε*_405_ (M^−1^·cm^−1^)	*ε*_450_ (M^−1^·cm^−1^)
OP1	370	12 500	4300	100
OP2	373	10 400	5100	800
OP3	371	12 100	4300	100

### Violet and Blue Light‐Induced Photopolymerization

**Figure 4:**
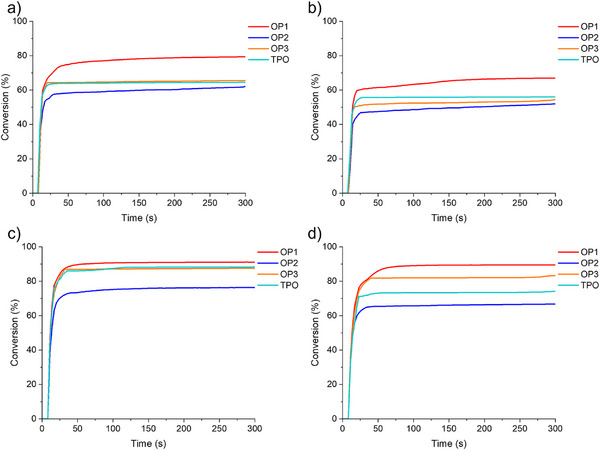
Photopolymerization kinetics of TMPTA or ETPTA with photoinitiators: a) PIs (2 × 10^−5^ mol·g^−1^ TMPTA), b) PIs (1 × 10^−5^ mol·g^−1^ TMPTA), c) PIs (2 × 10^−5^ mol·g^−1^ ETPTA), and d) PIs (1 × 10^−5^ mol·g^−1^ ETPTA) in laminate (thickness ∼28 µm) irradiated by LED@405 nm. Exposure starts at *t* = 10 s.

**Figure 5:**
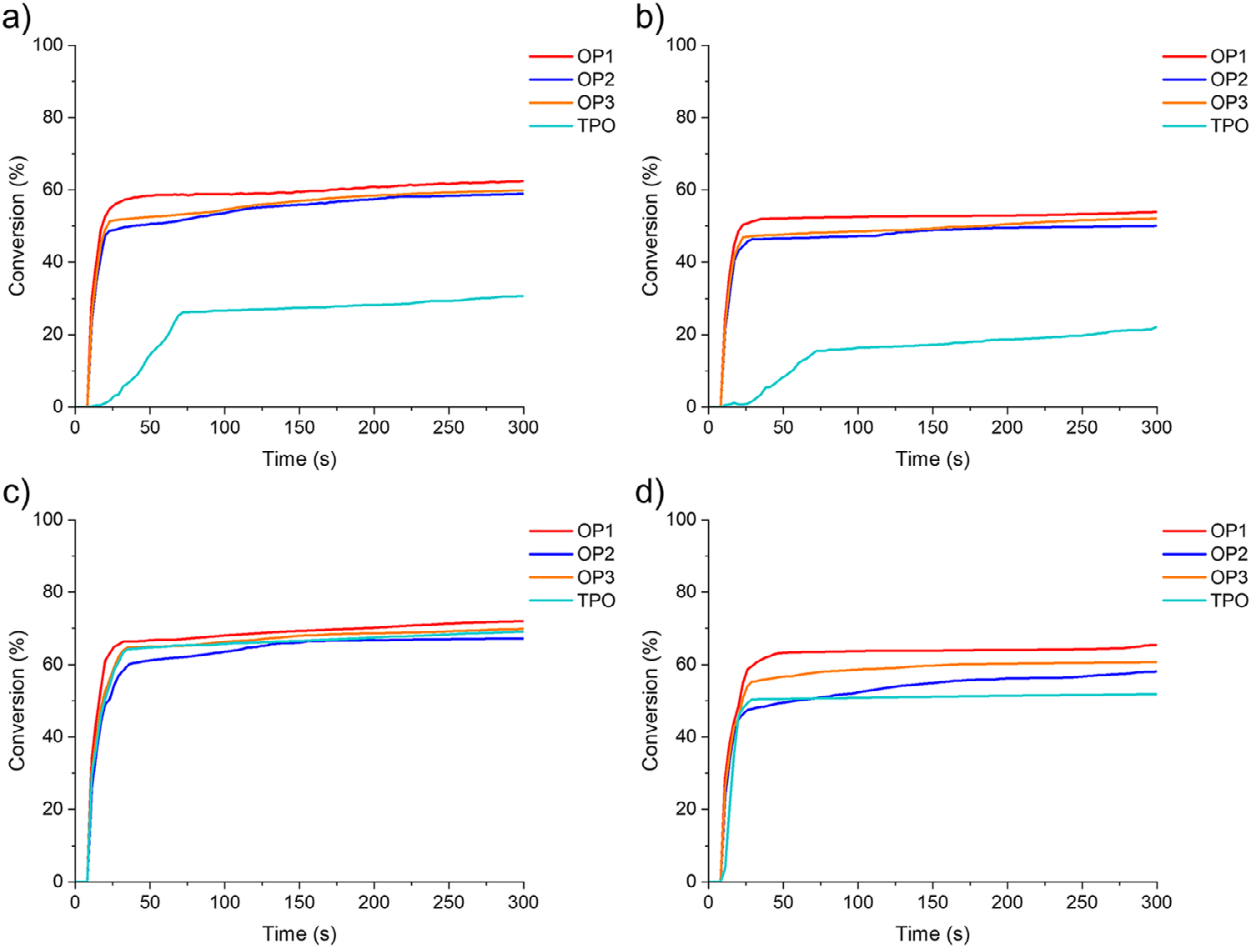
Photopolymerization kinetics of TMPTA or ETPTA with photoinitiators: a) PIs (2 × 10^−5^ mol·g^−1^ TMPTA), b) PIs (1 × 10^−5^ mol·g^−1^ TMPTA), c) PIs (2 × 10^−5^ mol·g^−1^ ETPTA), and d) PIs (1 × 10^−5^ mol·g^−1^ ETPTA) in laminate (thickness ∼28 µm) irradiated by LED@450 nm. Exposure starts at *t* = 10 s.

The photoinitiation capability of OPIs for the photopolymerization of TMPTA and ETPTA was investigated upon exposure to LED@405 nm (110 mW·cm^−2^) and LED@450 nm (50 mW·cm^−2^), while commercial photoinitiator TPO was used as control. Figures 4 and 5 present the kinetics of TMPTA and ETPTA photopolymerization initiated by different concentrations of PIs exposed to LED@405 nm and LED@450 nm. Table 4 summarizes the detailed results of the double bond conversions (Conv) in PIs/TMPTA and PIs/ETPTA. When the concentration of OPIs was 2 × 10^−5^ mol·g^−1^ monomer, OP1 and OP2 were completely soluble in TMPTA and ETPTA, whereas OP3 did not dissolve well in TMPTA and ETPTA. To avoid the effect of solubility on photoinitiation capability, the concentration was reduced to 1 × 10^−5^ mol·g^−1^ monomer, which allowed OPIs to dissolve completely in both TMPTA and ETPTA (See Figure S2 and Table S2). When the PIs concentration in TMPTA was 2 × 10^−5^ mol·g^−1^ TMPTA, the Conv of OP1/TMPTA was 79% exposed to LED@405 nm, which was greater than that of other OPIs/TMPTA and TPO/TMPTA (See Figure 4a). When the PIs concentration was reduced to 1 × 10^−5^ mol·g^−1^ TMPTA, the Conv of the corresponding PIs/TMPTA exposed to LED@405 nm occurred to decrease. However, OP1/TMPTA still had the highest Conv of 67% (See Figure 4b). The Conv of PIs/ETPTA also showed a similar trend to that of PIs/TMPTA under LED@405 nm irradiation (See Figure 4c,d). Markedly, the Conv of OP1/ETPTA was as high as 90% even for a photoinitiator concentration of 1 × 10^−5^ mol·g^−1^ ETPTA.

**Table 4:** The double bond conversions (Conv, expressed as %) of TMPTA and ETPTA with different concentrations of PIs exposed to LED@405 nm and LED@450 nm.

	2 × 10^−5^ mol·g^−1^ TMPTA		1 × 10^−5^ mol·g^−1^ TMPTA		2 × 10^−5^ mol·g^−1^ ETPTA		1 × 10^−5^ mol·g^−1^ ETPTA	
PIs	LED@ 405 nm	LED@ 450 nm	LED@ 405 nm	LED@ 450 nm	LED@ 405 nm	LED@ 450 nm	LED@ 405 nm	LED@ 450 nm
OP1	79	62	67	54	91	72	90	65
OP2	62	59	52	50	76	67	67	58
OP3	66	60	54	52	88	70	83	61
TPO	65	31	56	22	88	69	74	52

The Conv of OPIs/TMPTA and OPIs/ETPTA exposed to LED@450 nm (See Figure 5) showed a similar trend as exposed to LED@405 nm. Notably, OP1/TMPTA (Conv = 62%) and OP1/ETPTA (Conv = 72%) still presented the highest Conv under LED@450 nm irradiation (See Figure 5a,c). With decreasing PIs concentration, the Conv of OPIs/TMPTA and OPIs/ETPTA decreased (See Figure 5b,d). Importantly, the Conv of both OPIs/TMPTA and OPIs/ETPTA were higher than those of TPO/TMPTA and TPO/ETPTA. Significantly, the formulations stored at room temperature and protected from light were subjected to photopolymerization kinetics experiments under LED@405 nm after one month, and the results showed that OP1 could still exhibit a 73% high Conv (See Figure S3). It suggested that the formulations were well stabilized. Based on the above results, it might derive from the fact that a good solubility and monomers containing more flexible chain segments would result in better PI initiation properties. In summary, OP1 demonstrated better photoinitiation performance than other OPIs and the commercial photoinitiator TPO.

### Sunlight‐Induced Free Radical Photopolymerization

**Figure 6:**
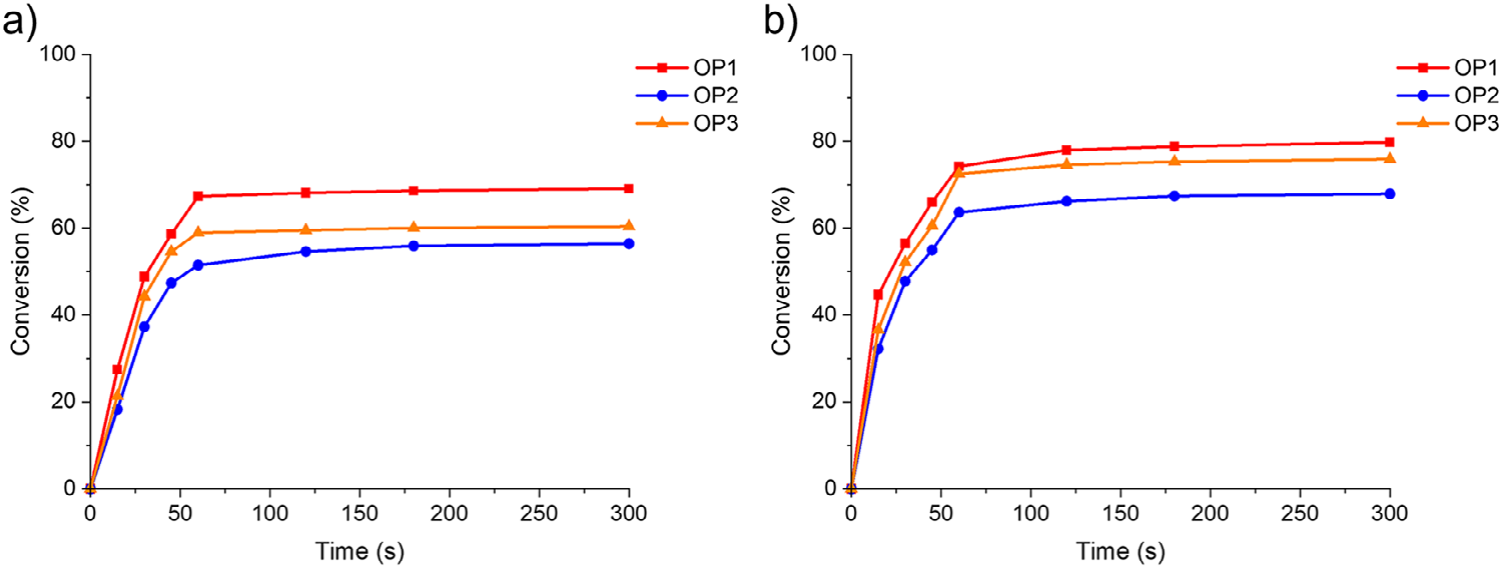
Photopolymerization kinetics of TMPTA or ETPTA with OPIs: a) 2 × 10^−5^ mol·g^−1^ TMPTA and b) 2 × 10^−5^ mol·g^−1^ ETPTA in laminate (thickness ∼28 µm) irradiated by sunlight. Exposure starts at *t* = 10 s.

Following the results of blue visible light‐induced photopolymerization experiments, the photoinitiation performance of OPIs (at a concentration of 2 × 10^−5^ mol·g^−1^ TMPTA and 2 × 10^−5^ mol·g^−1^ ETPTA) was investigated exposed to sunlight. The experimental location was set at 47°43′47′′ N and 7°18′32′′ E, and the weather was sunny. Figure S4 shows the detailed experimental weather conditions. OPIs exhibited good photoinitiation characteristics with high double bond conversions (Conv) under shorter sunlight exposure (See Figure 6 and Table 5). Remarkably, The Conv of OP1/TMPTA achieved 67% and 69% at 60 s and 300 s of light exposure, while those for OP1/ETPTA were even higher, at 75% and 80%, respectively. The results indicated that OP1 exhibited better photoinitiation capability compared to OP2 and OP3 under sunlight irradiation. Therefore, it can be concluded that OP1 demonstrated excellent prospects and promising applications in sunlight‐induced photopolymerization.

**Table 5:** The double bond conversions (Conv) of TMPTA and ETPTA with OPIs (2 × 10^−5^ mol·g^−1^ TMPTA and 2 × 10^−5^ mol·g^−1^ ETPTA) exposed to sunlight.

	PIs 2 × 10^−5^ mol·g^−1^ TMPTA			PIs 2 × 10^−5^ mol·g^−1^ ETPTA		
OPIs	60 s	120 s	300 s	60 s	120 s	300 s
OP1	67	68	69	74	78	80
OP2	51	55	56	64	66	68
OP3	59	59	60	73	75	76

### 3D Printing

**Figure 7:**
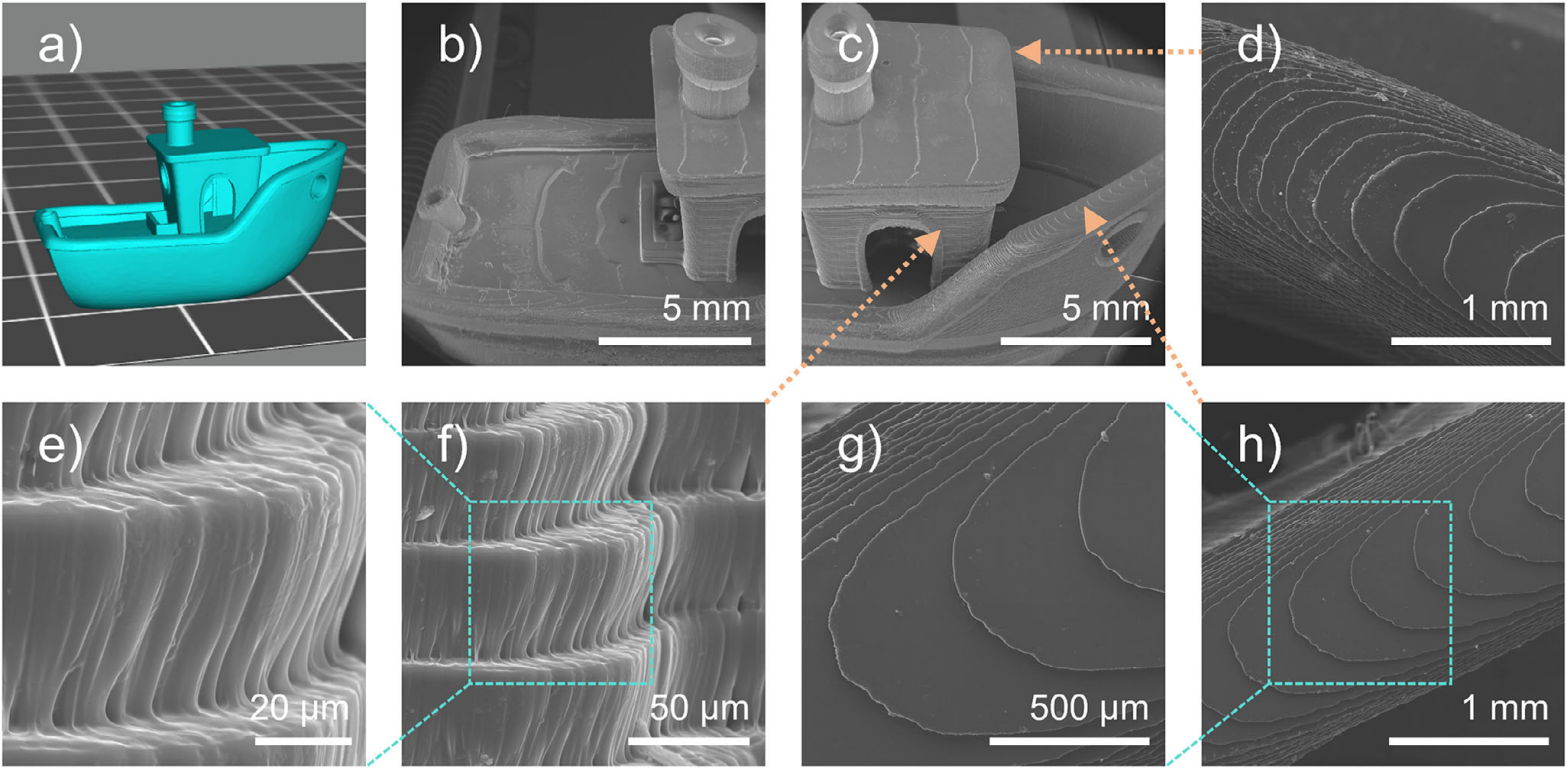
a) 3D boat model; b)–h) SEM images of the 3D object printed with OP1/TMPTA.

**Figure 8:**
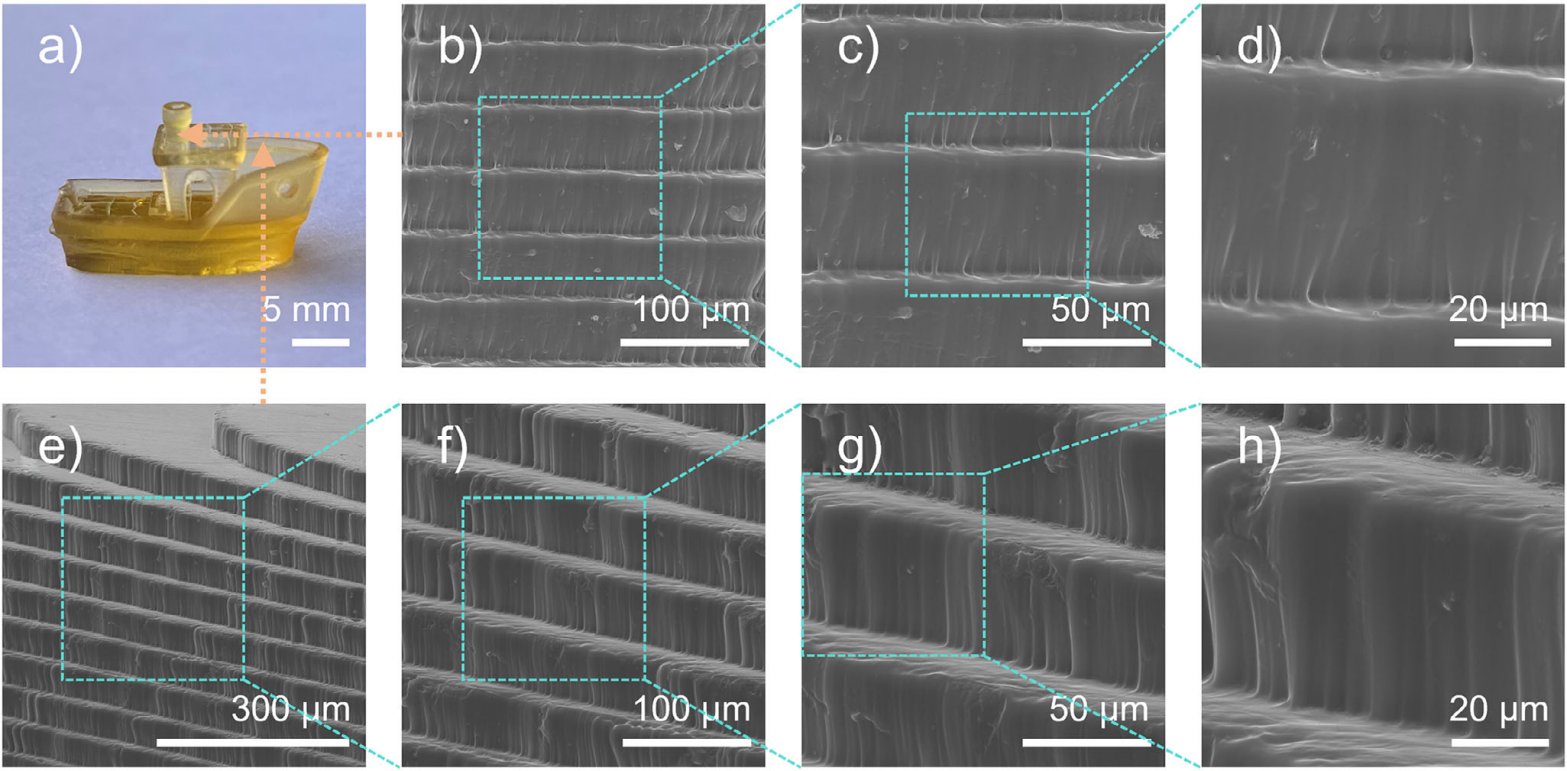
a) 3D object printed with OP1/TMPTA; b)–h) SEM images of the 3D object printed with OP1/TMPTA.

OP1 exhibited excellent photoinitiation performance in free radical photopolymerization, rendering it a desirable prospect for 3D printing. A representative 3D benchmark boat structure was fabricated using the OP1/TMPTA system employing a 3D printer (Anycubic Photon D2, 1.6 mW·cm^−2^ LED@405 nm) (See Figures 7 and 8). Scanning electron microscope (SEM) analysis of the printed object (top surface, outer side, and inner side) revealed a morphology that closely matched the digital model, with a smooth surface and well‐defined layer alignment along the print direction. A vertical resolution of 50 µm was achieved in the Z‐direction.

**Figure 9:**
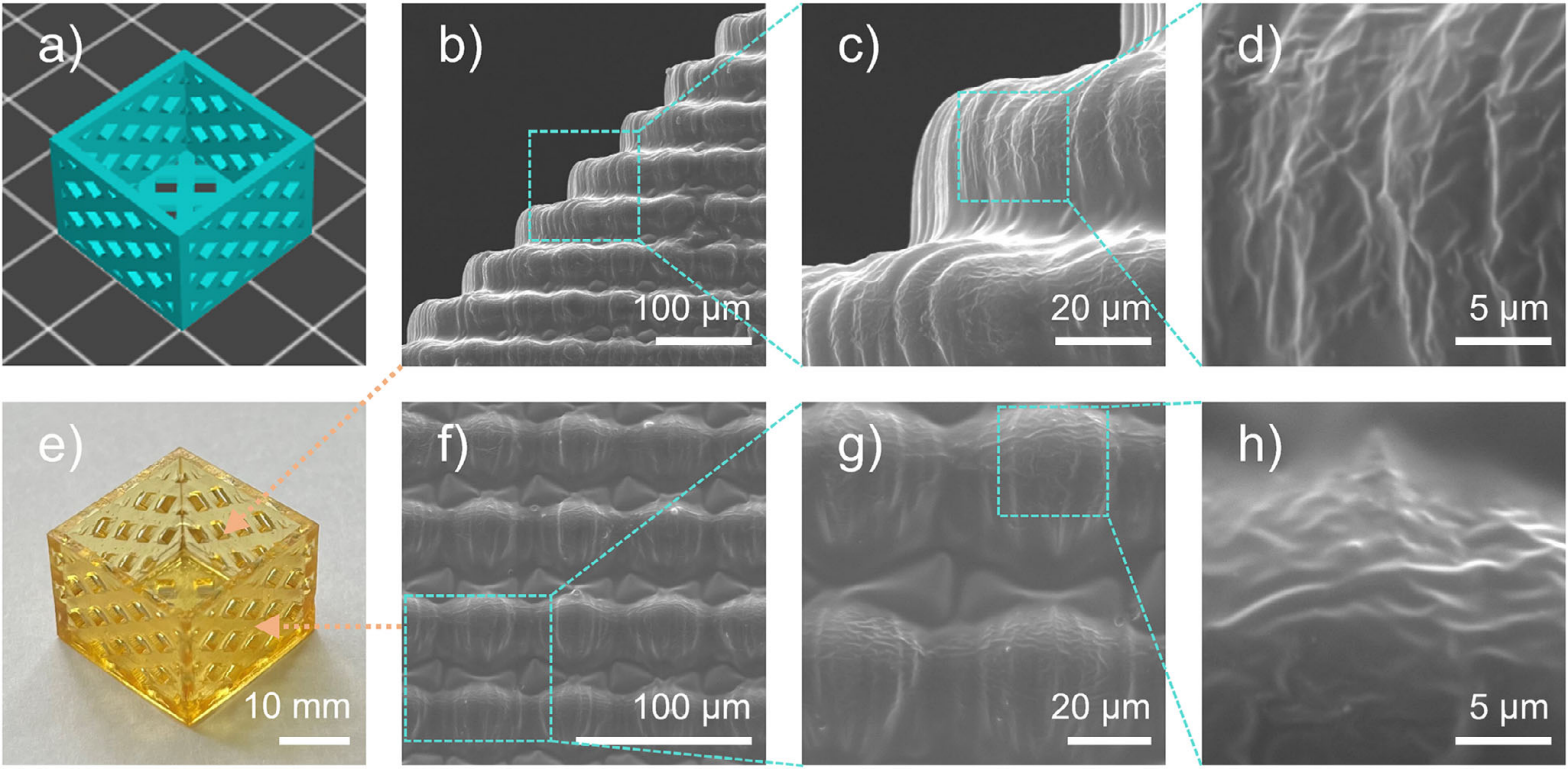
a) 3D mesh cubic box model; e) 3D object printed with OP1/ETPTA; b)–d) and f)–h) SEM images of the 3D object printed with OP1/ETPTA.

Furthermore, a mesh cubic box structure (20 mm × 20 mm × 15 mm) was printed using the OP1/ETPTA system and characterized by SEM (See Figure 9). The printed object exhibited regular, complete interlayer structures, clearly reflecting the layer‐by‐layer printing characteristics. A Z‐direction resolution of 25 µm was achieved, and morphological features remained discernible even at the 5 µm observation scale under SEM.

**Figure 10:**
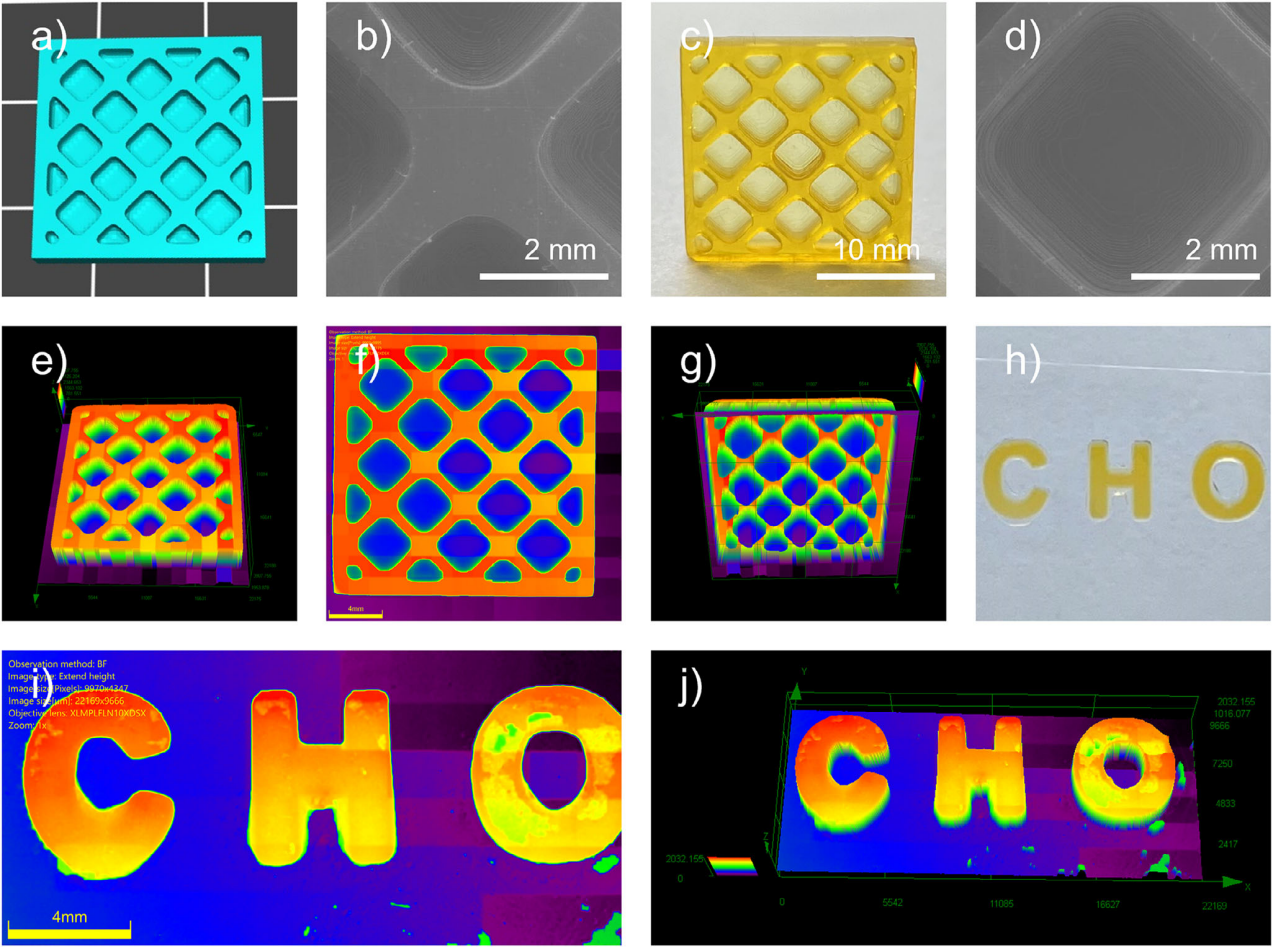
a) 3D lattice model; c) 3D object printed with OP1/TMPTA; b) and d) SEM images of the 3D object printed with OP1/TMPTA; e)–g) SEM images of the 3D object printed with OP1/TMPTA; h), one‐photon DLW patterns made with OP1/TMPTA (thickness = 2030 µm); i) and j) NOM images of the one‐photon DLW patterns made using OP1/TMPTA.

Additionally, OP1 was applied in DLP 3D printing and one‐photon direct laser writing (one‐photon DLW) (See Figure 10). A lattice object (20 mm × 20 mm × 4 mm) was successfully fabricated, followed by SEM and numerical optical microscope (NOM) characterization (See Figure 10a–g), which confirmed intact and well‐defined surface morphology. One‐photon DLW experiments were further conducted using a 405 nm laser diode, yielding patterns that maintained high morphological integrity (See Figure 10h–j). Collectively, these results demonstrated that OP1 enabled high‐efficiency 3D printing, underlining its potential for both academic research and practical applications.

### Curing Depth Working Curves of Photopolymer Resins

**Figure 11:**
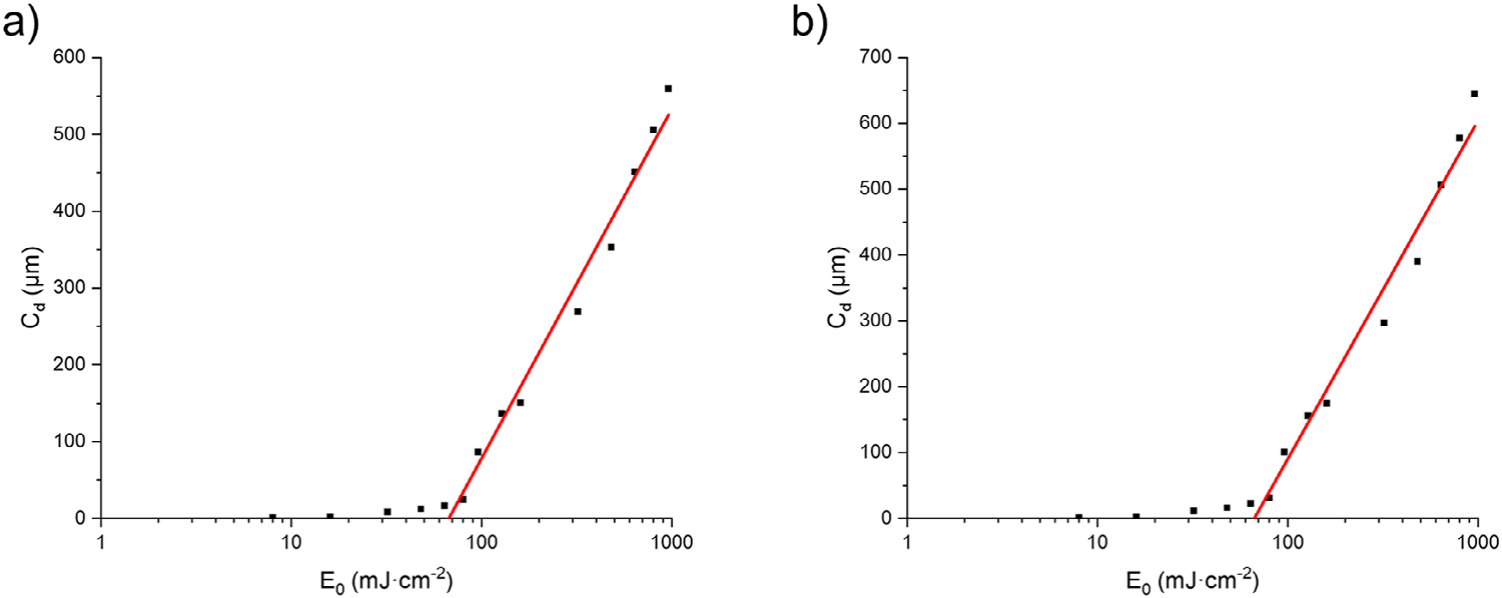
Curing depth working curves of a) OP1/TMPTA and b) OP1/ETPTA measured at a wavelength of 405 nm. *D*_p_ = 203 ± 10 µm and *E*_c_ = 71 ± 24 mJ·cm^−2^ were determined for the OP1/TMPTA system. *D*_p_ = 223 ± 12 µm, *E*_c_ = 67 ± 25 mJ·cm^−2^ were obtained for the OP1/ETPTA system.

To provide more comprehensive performance information for research in the field of 3D printing, the curing depth working curve (Jacobs working curve) analysis method was used in this study to elucidate light penetration characteristics and key energy requirements.^[^ ^53^, ^54^, ^55^ ^]^ Benchmark data were obtained using resin formulations containing only OP1/TMPTA and OP1/ETPTA (without additives). Exposure tests were conducted using a DLP 3D printer to determine the Jacobs working curves (See Figure 11) for the OP1/TMPTA and OP1/ETPTA systems. For the OP1/TMPTA system, a light penetration depth (D_p_) of 203 ± 10 µm and a critical enery exposure (*E*_c_) of 71 ± 24 mJ·cm^−2^ were determined. In contrast, the OP1/ETPTA system exhibited a light penetration depth of 223 ± 12 µm with a critical energy exposure of 67 ± 25 mJ·cm^−2^. Successful photocuring and 3D structure fabrication were achieved using radiant exposure of 80 mJ·cm^−2^ during 3D printing. These results not only matched the selected printing parameters but also provided valuable reference for optimizing photopolymer resin systems in 3D printing applications.

### Photochemistry of OPIs

#### Decarboxylation Mechanism

**Figure 12:**
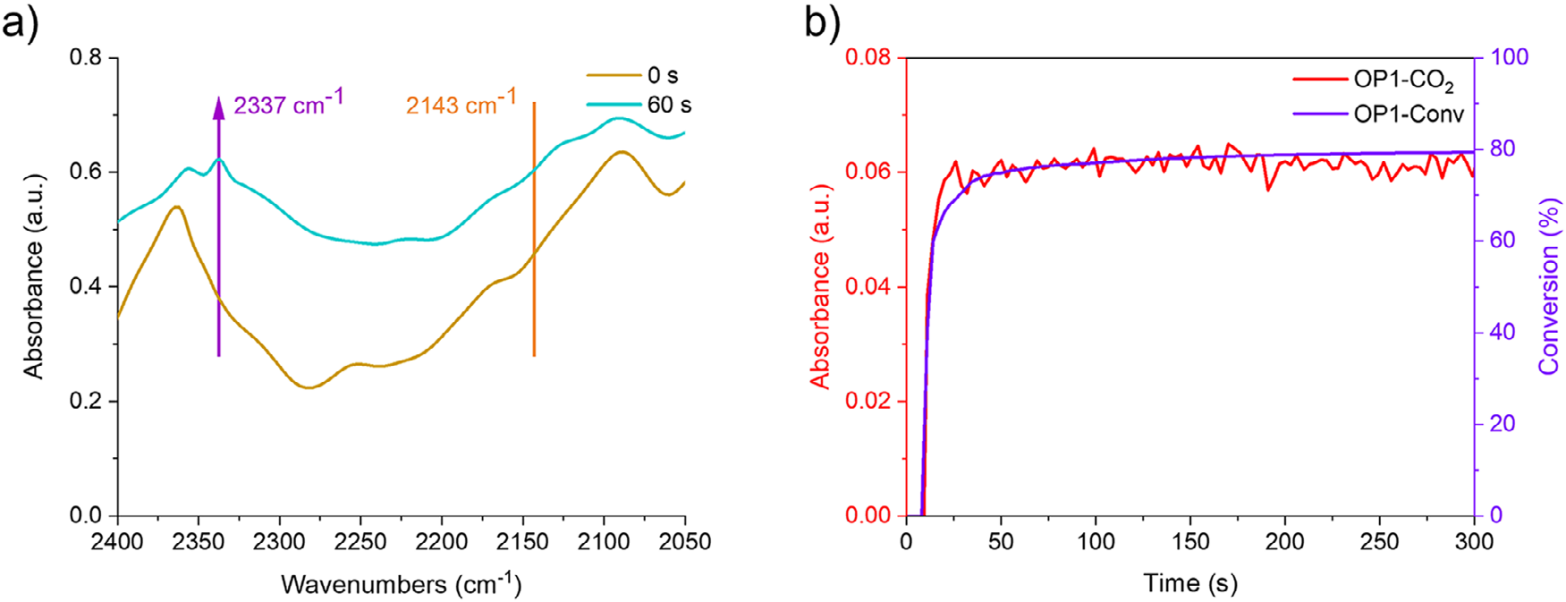
a) Infrared spectrum of OP1 in TMPTA at *t* = 0 s and 60 s. b) The curves of absorption intensity of CO_2_ released and double bond conversions (absorption intensity of CO_2_ and double bond conversions versus irradiation time) derived from OP1.

The occurrence of the decarboxylation reaction during the photopolymerization experiments was evaluated by detecting the production of CO_2_. The infrared spectrum of OP1 exhibited a prominent new absorption peak at 2337 cm^−1^ appearing during the photopolymerization process, which corresponded to the infrared absorption peak of CO_2_ (See Figure 12a).^[^ ^35^ ^]^ The structures of OPIs compounds influenced the extension of the decarboxylation reaction. Meanwhile, no CO infrared absorption peaks were found at 2143 cm^−1^ for OP1 and OP2 between *t* = 0 s and *t* = 60 s (See Figures 12a and S5a), which suggested that no significant amount of CO was produced during the photopolymerization process, i.e., no decarbonylation reaction occurred. This is consistent with the results predicted by molecular modeling. The CO_2_ production from OP1, OP3, and OP2 gradually decreased in the decarboxylation reaction (See Figure S5b), showing similar tendency to that of the progressive decrease of the photoinitiation performance of OP1, OP3, and OP2 during the photopolymerization reaction. The above results proved that OPIs underwent decarboxylation to generate CO_2_ and free radicals enabling the photopolymerization of monomers.

Furthermore, it was studied the relationship between the intensity of CO_2_ absorption, the double bond conversions, and the irradiation time (See Figure 12b). From *t* = 10 s to *t* = 20 s, the CO_2_ absorption intensity of OP1 increased rapidly, which was directly related to the CO_2_ produced during the photopolymerization process. After *t* = 60 s, the variation of CO_2_ absorbance was minimal. This observed trend coincided with the rapid increase of double bond conversions in OP1/TMPTA over the period of irradiation, suggesting that there existed a close relationship between CO_2_ production and the double bond conversions of monomers.

#### Steady State Photolysis

**Figure 13:**
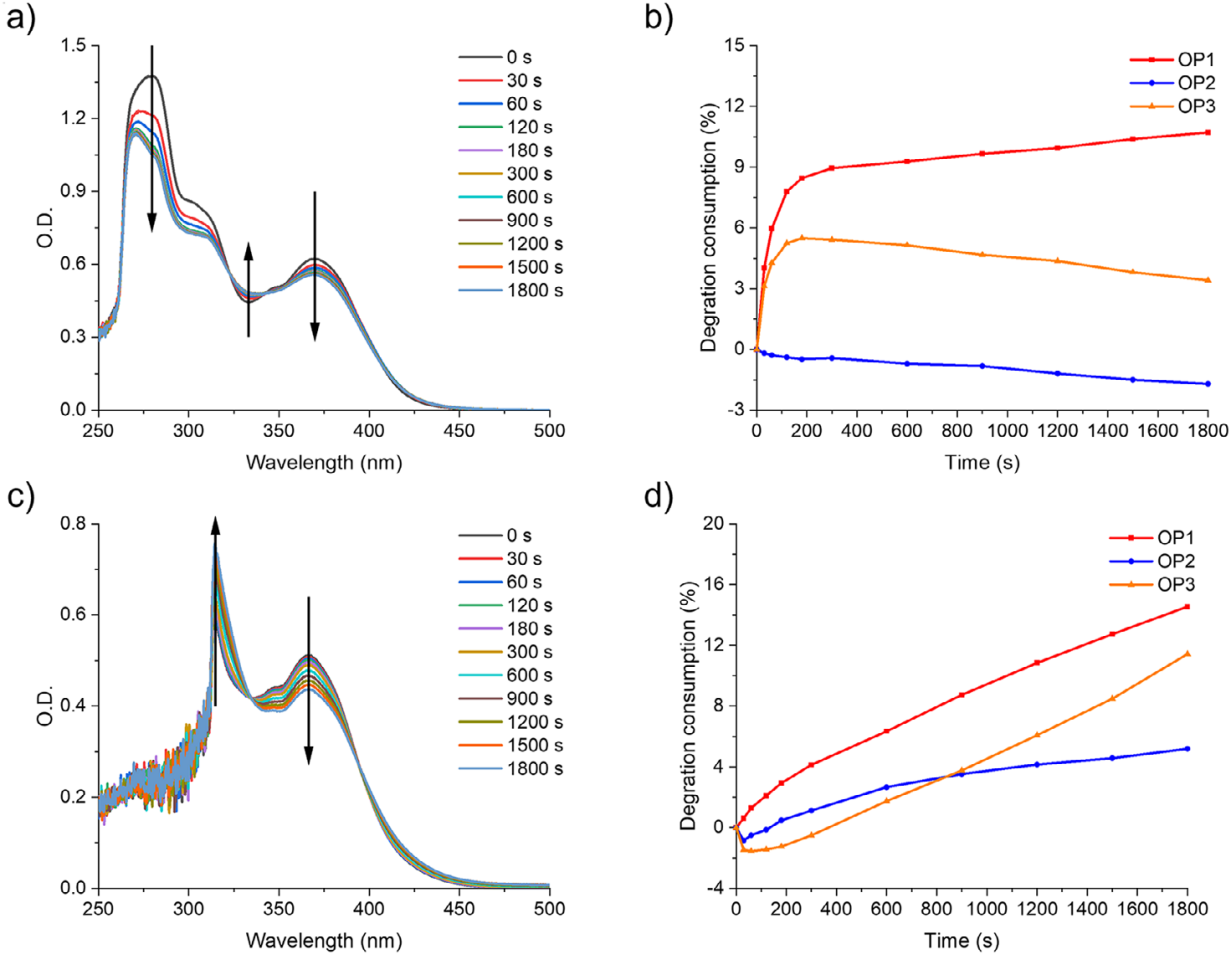
Steady state photolysis of OP1 in a) ACN and c) TMPTA exposed to LED@405 nm (concentration = 5 × 10^−5^ M). The curves of degradation rate and irradiation time from OPIs in b) ACN and d) TMPTA.

Steady state photolysis experiments were performed to investigate the photochemical mechanism of OP1 by solving in acetonitrile (ACN) or TMPTA under ambient air conditions (no oxygen removal) and irradiating it with LED@405 nm (See Figures 13a, S6, and S7). The intensity of the absorption peak of OP1 in acetonitrile was observed to decrease after 1800 s of light irradiation. Notably, two isosbestic points (∼320–330 nm and ∼340–350 nm) were found in the spectra (See Figure 13a), which clearly indicated that the cleavage process was carried out completely cleanly and no other side reactions occurred. However, OP1 showed the fastest degradation consumption rate and the largest amount of degradation consumption within 1800 s of irradiation time. (See Figure 13b). In order to minimize the effect of solvent cage effect, photolysis experiments were also carried out under the same conditions using TMPTA (See Figure S7). The intensity of the absorption peak of OP1 in TMPTA also decreased when exposed to LED@405 nm for 1800 s. And the isosbestic point (∼330–340 nm) also appeared in the spectra (See Figure 13c). OP1 remained the compound with the highest and fastest photolysis consumption (See Figure 13d). Combining the decarboxylation reaction and steady state photolysis experiments, it can be found that OP1 produced CO_2_ and free radicals for initiating the photopolymerization reaction of monomers.

#### ESR Experiments

**Figure 14:**
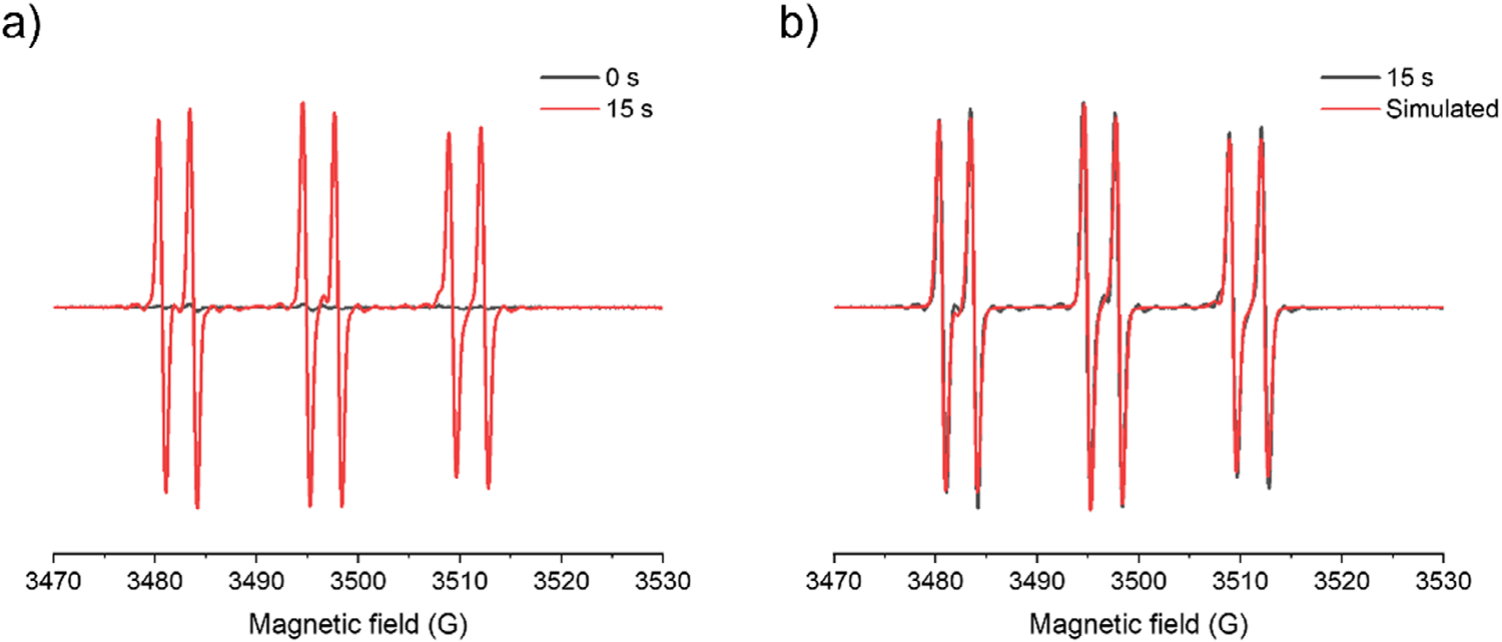
ESR spectra of a) OP1 radical adducts exposed to LED@405 nm captured by PBN in *tert*‐butylbenzene under N_2_ atmosphere before irradiation (black) and after 15 s (red) and b) superimposition of experimental and simulated ESR spectra of the methyl radical and PBN.

To further investigate the photochemical mechanism and the free radicals initiating the polymerization reaction, ESR experiment was carried out exposed to LED@405 nm with *n‐tert*‐butyl‐α‐phenylnitrone (PBN) as the radical trapping agent. The ESR spectra indicated that only one radical adduct of OP1 was detected, corresponding to the methyl radical in terms of the hyperfine coupling constants (HFCCs) of aN = 14.3 G and aH = 3.1 G (See Figure 14).^[^ ^56^ ^]^ Moreover, such clear signals and well‐resolved spectra obviously suggested that the cleavage reaction proceeded very cleanly and the free radicals were produced very rapidly (See Figure S8a). Because of this, OP1 showed high photoinitiation performance in sunlight‐induced photopolymerization. These results aligned well with both molecular modeling prediction and the high initiation performance of OP1 in the photopolymerization experiments. Based on the experimental results of decarboxylation and ESR, it can be suggested that the decarboxylation reaction occurred during the photopolymerization reaction, i.e., the N─O, C─C, and C─O bonds of OPIs were inevitably cleaved. Therefore, the cleavage reaction could still occur despite the positive values of the singlet state cleavage enthalpy (C─C and C─O cleavage for S1, Δ*H*_cleavage S1_ > 0).

In addition, ESR experiments were also performed on OP2. The ESR spectra results revealed three radical adducts of OP2, with HFCCs of aN = 13.4 G and aH = 1.7 G; aN = 14.7 G and aH = 3.3 G; and aN = 14.2 G and aH = 3.0 G, respectively (See Figure S8b,c). The first radical adduct corresponded to the oxygen‐centered radical formed after N─O bond cleavage, while the latter two adducts corresponded to the acyl radical generated following the cleavage of the dicarbonyl moiety. This result was consistent with molecular modeling and decarboxylation reaction, indicating that OP2 produced carbon dioxide rather than carbon monoxide.

#### Photoinitiation Mechanism

**Scheme 3:**
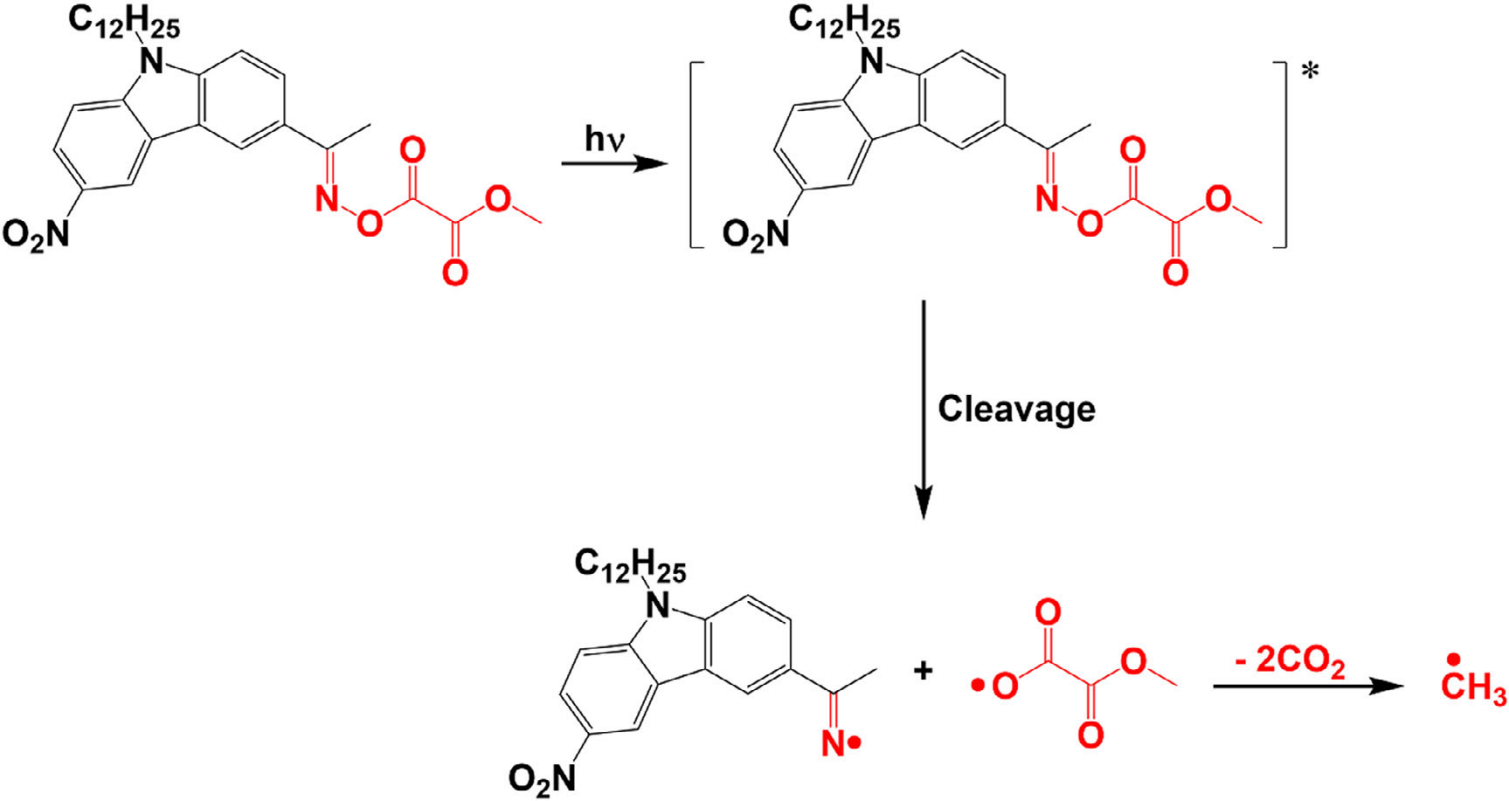
Proposed photoinitiation mechanisms of OP1.

With the results described above, the proposed potential photoinitiation mechanism for OP1 is presented in Scheme 3. The N─O bond of OP1 cleaved first to generate iminyl radical and oxalate radical exposed to LED@405 nm. Subsequently, the C─C and C─O bonds in the oxalate moiety cleaved, and the decarboxylation reaction proceeded rapidly to generate CO_2_ and methyl radicals, which initiated the polymerization of the acrylate monomers. Such a simple initiation mechanism could efficiently generate abundant methyl radicals to initiate the polymerization reaction. This finding contributes significantly to the design of Type I photoinitiators with oxime oxalate moiety, and is favorable for the exploration and preparation of new Type I photoinitiators with high initiation performance.

### Thermal Polymerization Experiments

**Figure 15:**
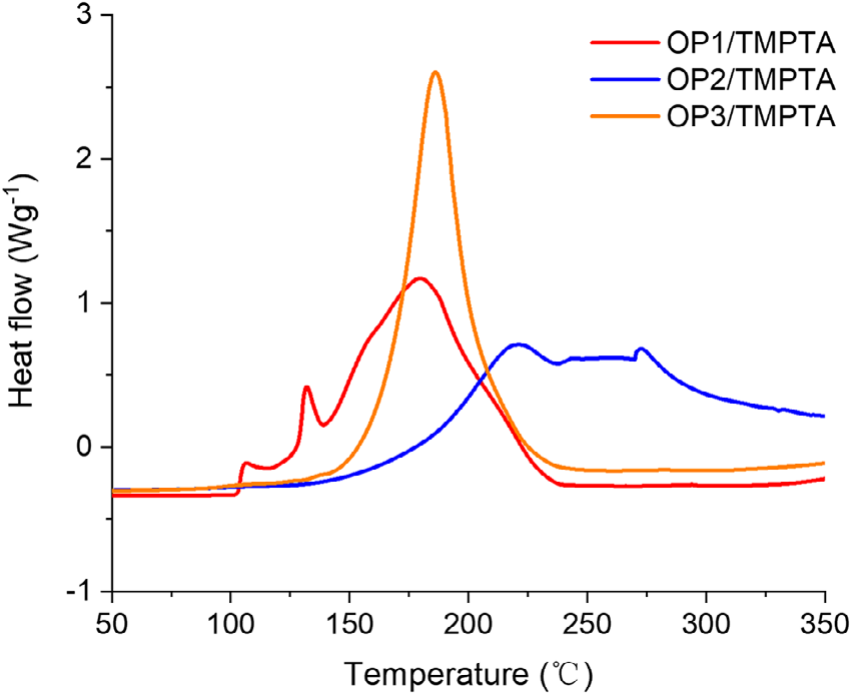
DSC curves of OPIs/TMPTA.

**Figure 16:**
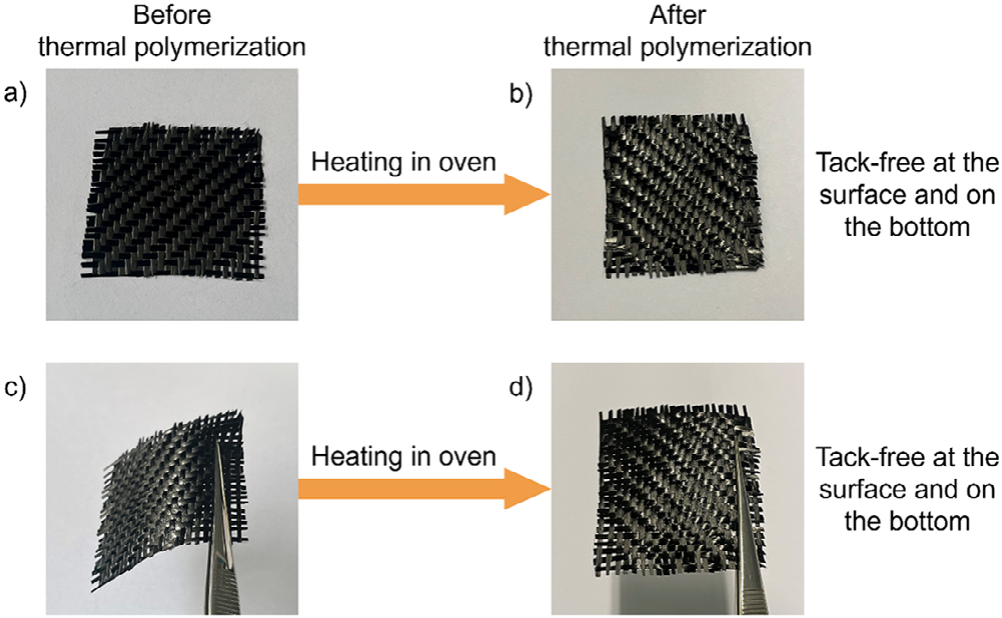
a) and c) Prepreg prepared from OP1/TMPTA and carbon fibers (50%/50% w/w) before heating; b) and d) thermal composite prepared from OP1/TMPTA and carbon fibers (50%/50% w/w) after heating. The prepreg underwent gravitational deformation before heating c), while the thermal composite maintained structural integrity under the same conditions after heating d).

Figure 15 and Table 6 show that the thermal polymerization initial temperature (T_initial_) of OPIs/TMPTA (2 × 10^−5^ mol·g^−1^ TMPTA) were all above 80 °C, and the temperature of maximum polymerization rate (T_max_) were all higher than 170 °C. This indicated that OPIs had good thermal stability and high tolerance to ambient temperature. The thermal initiation property of OP1 was subsequently investigated by thermal composite prepared by prepreg of carbon fibers and OP1/TMPTA (50%/50% w/w) (See Figure 16). The TMPTA alone remained fluid after heating in an oven at 150 °C for 30 min.^[^ ^57^ ^]^ The prepreg was easily deformed before heating (See Figure 16a,c), while the composite prepared after heating was hard and resistant to deformation (See Figure 16b,d). The results suggested that the prepreg underwent deep thermal polymerization after 30 min of heating in an oven at 140 °C and achieved complete curing. The surface and bottom of the composite were fully cured, smooth, and tack‐free. In addition, OP1/TMPTA exhibited high double bond conversions (Conv = 69%), which was higher than that of TMPTA alone (Conv = 44%).^[^ ^11^ ^]^ Thus, OP1 demonstrated excellent thermal initiation capability, which was consistent with the results predicted by molecular modeling–OP1 possessed a lower decarboxylation reaction energy barrier and was therefore more likely to generate free radicals that could initiate the polymerization reaction of the monomer.

**Table 6:** Thermal polymerization of OPIs/TMPTA.

OPIs/TMPTA	*T*_initial_ (°C)	*T*_max_ (°C)	Conv (%)
OP1/TMPTA	88	180	69
OP2/TMPTA	118	220	60
OP3/TMPTA	107	187	65

### Cytotoxicity Experiments

**Figure 17:**
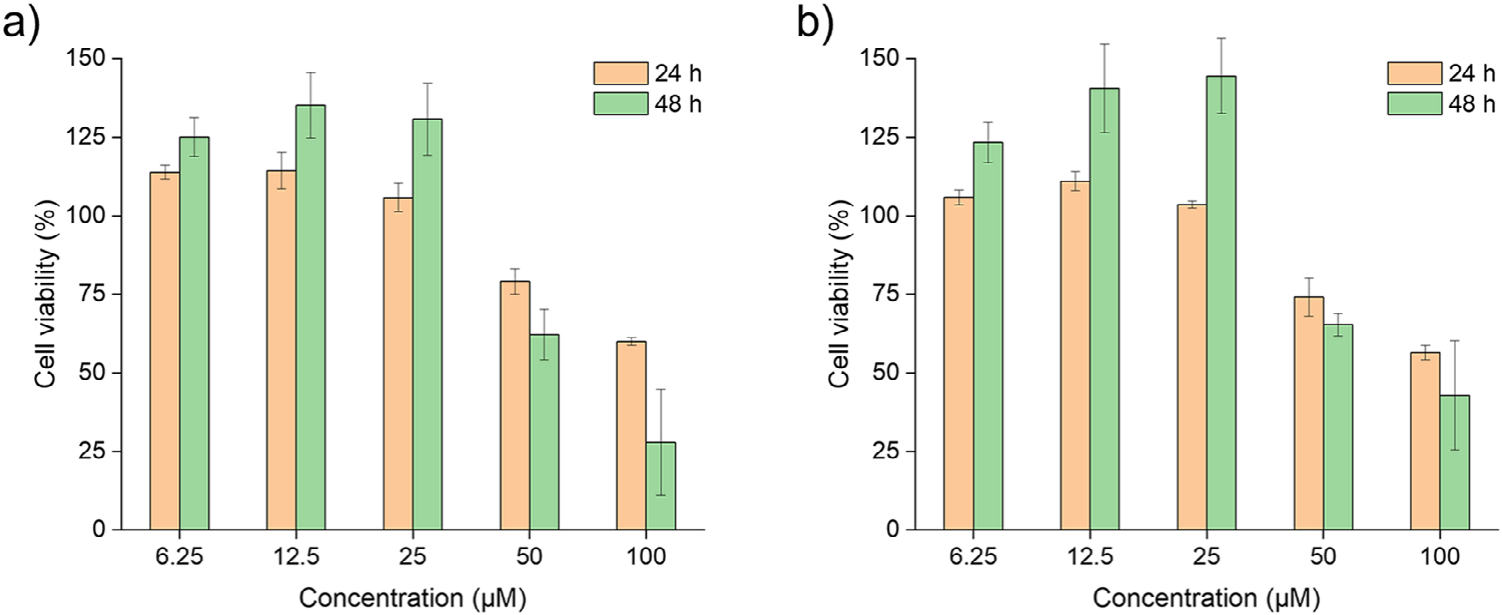
Cytotoxicity of different concentrations of a) light TPO treated and b) light OP1 treated groups in HUVECs after 24 h and 48 h of incubation.

With the widespread research and use of photoinitiators, their cytotoxicity has attracted increasing attention. In cytotoxicity experiments, the common concentration of photoinitiator used was 50 µM, and the duration of the experiment was usually 24 h.^[^ ^58^, ^59^ ^]^ In this study, the photoinitiator concentration was increased to 100 µM, the duration of the experiment was prolonged to 48 h, and both experimental conditions with and without light were used (light TPO/OP1 treated and no‐light TPO/OP1 treated indicated with and without light), aiming to systematically reveal the effects of the photoinitiator concentrations and light on the cytotoxicity, so as to obtain a more comprehensive and in‐depth assessment of biocompatibility. The cytotoxicity of OP1 in human umbilical vein endothelial cells (HUVECs) was evaluated using the Cell Counting Kit‐8 (CCK‐8) assay with TPO as control (See Figure 17).

The results showed that the cytotoxicity of OP1 was significantly lower than that of TPO at a concentration of 100 µM and under light conditions, specifically, the cell viability of the light OP1 treated group was 56% and 43% at 24 and 48 h, respectively, compared with that of the light TPO treated group, which was 60% and 28%. In addition, under other concentration conditions, the cell viability of the light OP1 treated group was generally higher than that of the light TPO treated group at 48 h, which further verified that OP1 had better cytocompatibility. In the absence of light, OP1 also showed low toxicity, which was demonstrated by higher cell viability in the no‐light OP1 treated group than in the no‐light TPO treated group at a concentration of 100 µM at 48 h (See Figure S9). The above results comprehensively indicated that OP1 exhibited low toxicity and biosafety under both light and no light conditions. Therefore, OP1 as a dual photo/thermal initiator shows promising potential for future applications.

## Conclusions

In this work, we designed three nitro carbazole‐based oxime derivatives (OPIs), namely oxime oxalate (OP1), oxime glyoxylate (OP2), and oxime ester (OP3). In contrast to our earlier study on glyoxylate oxime ester, the present work expanded the design space by introducing oxime oxalate, which exhibited a distinct initiation mechanism and broader application scope, thereby providing a novel and versatile platform for visible‐light and thermal polymerization. The light absorption properties, photoinitiation properties, and decarboxylation of the OPIs were first predicted by molecular modeling. Using molecular modeling, it was first predicted that the OPIs compounds containing these structures had good light absorption characteristics and all of them could undergo decarboxylation, with OP1 having the best photoinitiation performance. The light absorption study demonstrated that these OPIs compounds exhibited light absorption properties at both 405 and 450 nm. The photopolymerization kinetics experiments showed that the photoinitiation performance of OP1 was better than that of commercial photoinitiator TPO exposed to LED@405 nm and LED@450 nm. Remarkably, OP1 also showed excellent photoinitiation capability in sunlight‐induced photopolymerization as well as in 3D printing. The photochemical mechanism of OP1 was comprehensively analyzed by monitoring the decarboxylation reaction, steady state photolysis, cleavage enthalpy, and ESR. The photoinitiation mechanism suggested that OP1 rapidly underwent decarboxylation under LED@405 nm irradiation, generating CO_2_ and large quantities of methyl radicals accessible for initiating photopolymerization reactions. Interestingly, OP1 still showed good initiation performance in thermal polymerization experiments. It indicated that OP1 was an efficient dual photo/thermal initiator. Finally, it was assessed by CCK‐8 assay that OP1 with and without light‐treated had low cytotoxicity, which could be a good indicator of the safety of OP1 in biomedical applications. Significantly, it was employed for the first time in this study to explore the properties of OPIs by molecular modeling first. The feasibility and reliability of the molecular modeling theoretical calculations were subsequently verified experimentally for realism. The strategy of combining theoretical calculations followed by experiments adopted in this research provides a new direction and idea for the preparation of novel and efficient Type I PIs, which has a broad development prospect.

## Author Contributions

**T.G**.: Conceptualization; data curation; formal analysis; investigation; methodology; validation; writing—original draft; Review & editing. **T.D.M**.: Formal analysis; writing—review & editing. **J.F**.: Review & editing; **J.Z**.: Formal analysis; writing—review & editing. **F.M.S**.: Formal analysis; supervision;. **C.D**.: Software; review & editing. **M.S**.: Review & editing. **F.D**.: Formal analysis; review & editing. **J.P.J**.: Review & editing. **M.N**.: Conceptualization; formal analysis; writing—review & editing. **P.X**.: Supervision; formal analysis; writing—review & editing. **J.L**.: Funding acquisition; project administration; resources; supervision; validation; writing—review & editing.

## Conflict of Interests

The authors declare no conflict of interest.

## Supporting information



Supporting Information

## Data Availability

The data that support the findings of this study are available from the corresponding author upon reasonable request.
