# Supplementary material for: Structure–Reactivity Relationships of Oxime–Oxalate/Glyoxylate/Ester Derivatives: Dual Photo/Thermal Initiators for Visible Light Polymerization and Composite Preparation
Source: Angew Chem Int Ed Engl. 2025 Nov 30;65(4):e21296. doi: 10.1002/anie.202521296 (PMC12828456; doi:10.1002/anie.202521296)
Supplement: Supplementary file 1 — Supporting Information [file ANIE-65-e21296-s001.docx]

**Structure–Reactivity Relationships of Oxime–Oxalate/Glyoxylate/Ester Derivatives: Dual Photo/Thermal Initiators for Visible Light Polymerization and Composite Preparation**

Tong Gao ^a,b,c^, Thybault De Monfreid ^d^, Ji Feng ^b,c^, Jing Zhang ^e^, Fabrice Morlet-Savary ^b,c^, Céline Dietlin ^b,c^, Michael Schmitt ^b,c^, Frédéric Dumur ^d^, Jean-Patrick Joly ^d^, Malek Nechab ^d^*, Pu Xiao ^a^*, and Jacques Lalevée ^b,c^*

^a^ State Key Laboratory of High Performance Ceramics, Shanghai Institute of Ceramics, Chinese Academy of Sciences, Shanghai 200050, P. R. China.

^b^ Université de Haute-Alsace, CNRS, IS2M UMR7361, F-68100 Mulhouse, France.

^c^ Université de Strasbourg, France.

^d^ Aix Marseille Univ, CNRS, ICR, UMR 7273, F-13397 Marseille, France.

^e^ Future Industries Institute, University of South Australia, Mawson Lakes, SA 5095, Australia.

E-mail address: [jacques.lalevee@uha.fr](mailto:jacques.lalevee@uha.fr) (JL), [p.xiao@mail.sic.ac.cn](mailto:p.xiao@mail.sic.ac.cn) (PX), [malek.nechab@univ-amu.fr](mailto:malek.nechab@univ-amu.fr) (MN)

**Supporting Information**

**Table of Contents**

[1. Experimental Sections S2](#_Toc209885571)

[1.1 Computational procedures S2](#_Toc209885572)

[1.2 Materials S2](#_Toc209885573)

[1.3 UV-visible absorption property and steady state photolysis experiments S2](#_Toc209885574)

[1.4 Free radical photopolymerization experiments S2](#_Toc209885575)

[1.5 Fluorescence property experiments S2](#_Toc209885576)

[1.6 Electron spin resonance spin trapping (ESR-ST) experiments S2](#_Toc209885577)

[1.7 3D printing experiments S3](#_Toc209885578)

[1.8 Jacobs working curve experiments S3](#_Toc209885579)

[1.9 Thermal polymerization experiments S3](#_Toc209885580)

[1.10 Cytotoxicity assays S4](#_Toc209885581)

[2. Supplementary Figures S5](#_Toc209885582)

[3. Supplementary Tables S11](#_Toc209885583)

[4. General information S12](#_Toc209885584)

[5. Reference S17](#_Toc209885585)

1. Experimental Sections

1.1 Computational procedures

Computational molecular modeling of OPIs was performed using the Gaussian 09 software package. Optimizations were conducted at the B3LYP/6-31G* level of theory. Orbitals were optimized at the MPW1PW91/6-31g(d) level of theory at a single point.

1.2 Materials

Acetonitrile (ACN), *tert*-butylbenzene, and *n-tert*-Butyl-α-phenylnitrone (PBN) were obtained from Sigma-Aldrich. Trimethylolpropane triacrylate (TMPTA), ethoxylated trimethylolpropane triacrylate (ETPTA), and 2,4,6-trimethyl(phenyl) diphenyl oxide (TPO) were procured from Sartomer.

1.3 UV-visible absorption property and steady state photolysis experiments

The UV-visible absorption properties, molar extinction coefficients and steady state photolysis of OPIs (5 × 10^-5^ M) in ACN were investigated using a JASCO V730 UV-visible spectrometer provided with a LED@405 nm (110 mW·cm^-2^) radiation source.

1.4 Free radical photopolymerization experiments

The photopolymerization kinetics of OPIs/TMPTA and OPIs/ETPTA was evaluated using a JASCO FTIR-6600 spectrometer equipped with LED@405 nm (110 mW·cm^-2^) and LED@450 nm (50 mW·cm^-2^). Mixtures of OPIs/TMPTA and OPIs/ETPTA (2×10^-5^ mol·g^-1^ and 1×10^-5^ mol·g^-1^ in TMPTA and ETPTA) was stirred for 24 h under dark conditions, and then polymerization solution was transferred in laminates for photopolymerization experiments. The double bond conversions (Conv) were calculated by monitoring the signal area of the polymerization solution at approximately 1620 cm^-1^. The following equation was used to calculate the Conv of TMPTA and ETPTA:

$$Conv\left( \% \right)=\frac{A_{0}-A_{t}}{A_{0}}\times100\%$$

where A_0_ indicates the peak area at 0 s, and A_t_ indicates the peak area at t s.

1.5 Fluorescence property experiments

The fluorescence properties of OPIs (5 × 10^-5^ M) in ACN were investigated using a JASCO FP-6200 spectrofluorometer. The fluorescence lifetimes of OPIs were measured with the assistance of the time-correlated single-photon counting system HORIBA® DeltaFlex and the HORIBA® PPD-850 detector. The impulse response function (IRF) of the instrument was evaluated by means of a colloidal silica suspension LUDOX.

1.6 Electron spin resonance spin trapping (ESR-ST) experiments

An X-band spectrometer (Bruker EMX-Plus) with LED@405 nm light source was employed to detect free radicals. The ESR-ST experiments were performed at room temperature under N_2_ atmosphere with a concentration of 1×10^-4^ M for OPIs. PBN (5×10^-4^ M) and *tert*-butylbenzene were used as radical trapping agent and solvent, respectively. And the ESR spectra were simulated using the PEST WINSIM program.

1.7 3D printing experiments

The preparation procedures for the 3D printing inks were conducted identically to the preparation of polymerization solution in photopolymerization experiments, with no other additives incorporated. The photoinitiator concentration was 2×10^-5^ mol·g^-1^ TMPTA or ETPTA. OP1/TMPTA and OP1/ETPTA were 3D printed using a 3D printer (Anycubic Photon D2, 1.6 mW·cm^-2^ LED@405 nm) derived from digital light processing (DLP) light-curing molding technology. The benchmark boat model, mesh cubic box model, and lattice model were used in the DLP 3D printing experiments. The slice thicknesses were 0.05 mm, 0.025 mm, and 0.03 mm, respectively, with an irradiation time of 50 s per layer. The 3D printed object was characterized using scanning electron microscope (SEM, FEI QUANTA 400) and numerical optical microscope (NOM, OLYMPUS DSX-HRSU). One-photon direct laser writing (one-photon DLW) experiments were conducted using NEJE diode Laser @405 nm (spot size ~ 50 μm). The objects gained in DLW was visualized with NOM.

1.8 Jacobs working curve experiments

The Jacobs working curve was performed using a 3D printer (Anycubic Photon D2, 1.4 mW·cm^-2^ LED@405 nm) derived from digital light processing (DLP) light-curing molding technology. First, the light intensity of the printer was calibrated sensor USB power meter (Thorlabs PM16-425) to obtain accurate light energy density. Subsequently, 3D printing inks (OP1/TMPTA and OP1/ETPTA) containing only photoinitiator and monomer (without other additives) were prepared. The photoinitiator concentration was 2×10^-5^ mol·g^-1^ TMPTA or ETPTA. Under varying light energy density conditions, inks were quantitatively exposed via the 3D printer, with each condition repeated at least three times to ensure data reliability. The thickness of cured samples was measured using mitutoyo measuring instruments, and the corresponding light energy density was recorded. Finally, the measured thickness C_d_ and exposure energy E_0_ were substituted into the Jacobs equation for fitting:

$$C_{d}\left( E_{0} \right) =D_{p} ln\left( \frac{E_{0}}{E_{C}} \right)$$

where C_d_ represents the curing depth of the ink, E_0_ denotes the dose exposure, D_p_ signifies the light penetration depth, and E_c_ indicates the critical energy exposure required to convert liquid ink into a solid state.

1.9 Thermal polymerization experiments

The thermal initiation and thermal stability properties of OPIs (2×10^-5^ mol·g^-1^ TMPTA) were investigated using Mettler-Toledo differential scanning calorimetry (DSC) under N_2_ ambient. The double bond conversions (Conv) of acrylate functional groups were calculated employing the following equation:

$$Conv\left( \% \right)=\frac{Heat released}{795.9}\times100\%$$

The thermal composites were then prepared by coating the carbon fibers with organic resin (carbon fibers/organic resin 50%/50% w/w). Next, the prepreg was heated in the oven. Thermal polymerization capabilities of the OPIs were measured.

1.10 Cytotoxicity assays

A 1 mM solution of photoinitiators (PIs) was prepared with dimethyl sulfoxide (DMSO). Then, it was diluted with Dulbecco’s modified Eagle’s medium/F12 (DMEM/F12) containing 10 % fetal bovine serum (FBS) to different concentrations (6.25, 12.5, 25, 50 and 100 μM). The PIs solutions were irradiated with or without LED@405 nm for 5 min as light treated and no-light treated groups, respectively. Human umbilical vein endothelial cells (HUVECs) were seeded in DMEM/F12 containing 10 % FBS at a density of 1 × 10^4^ cells/well into 96-well plates and cultured overnight in humidified incubator (37 °C, 5 % CO_2_). HUVECs cells were co-cultured with medium containing different concentrations of PIs for 24 h and 48 h. The cytotoxicity of photoinitiators in HUVECs was evaluated using Cell Counting Kit (CCK-8, Beyotime Biotechnology). The reagents for the cell experiment were acquired from Gibco.

2. Supplementary Figures


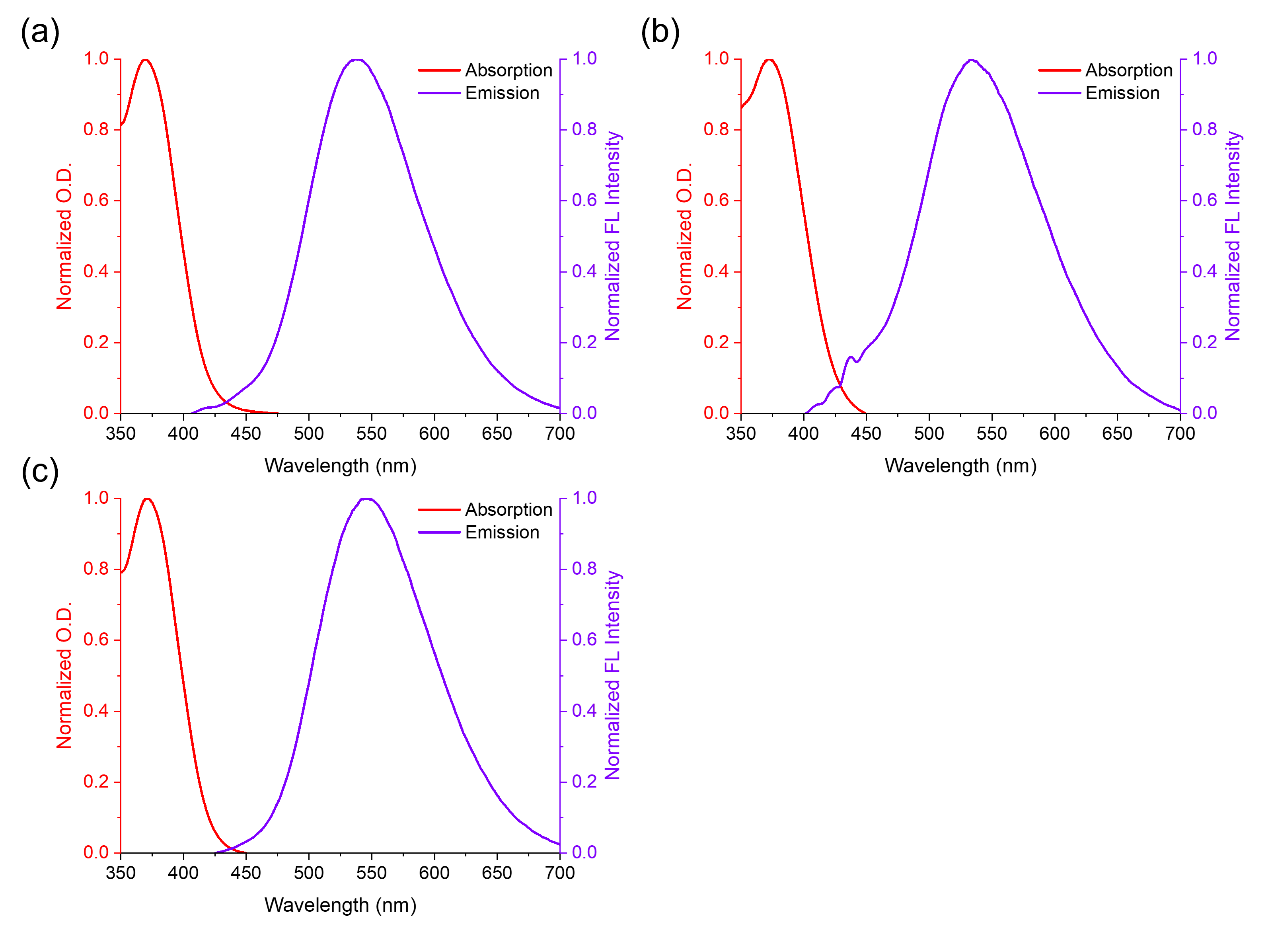


**Figure S1.** The curves of singlet-state energy determination of (a) OP1, (b) OP2, and (c) OP3.


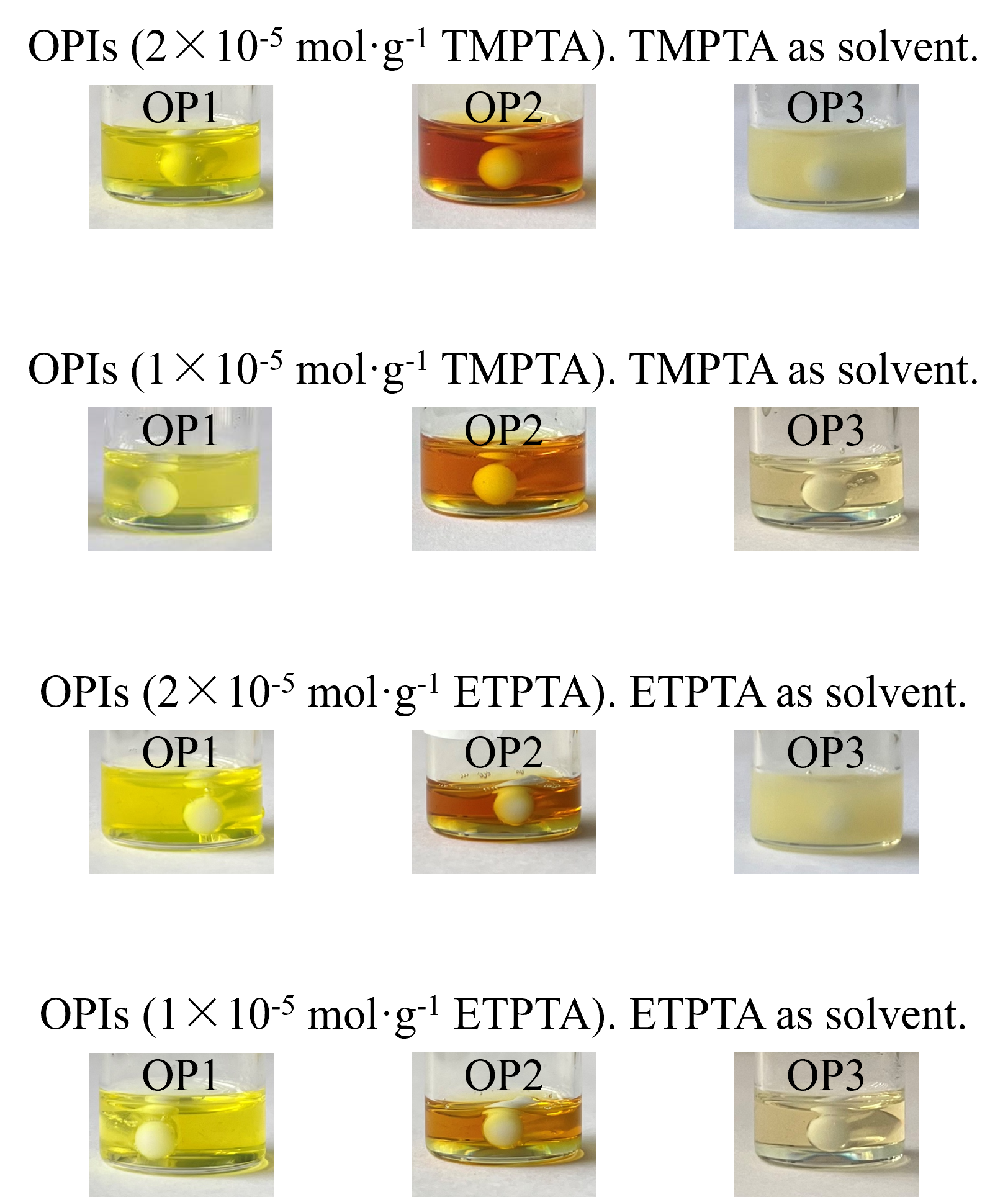


**Figure S2.** Solubility of OPIs. TMPTA and ETPTA as solvent.


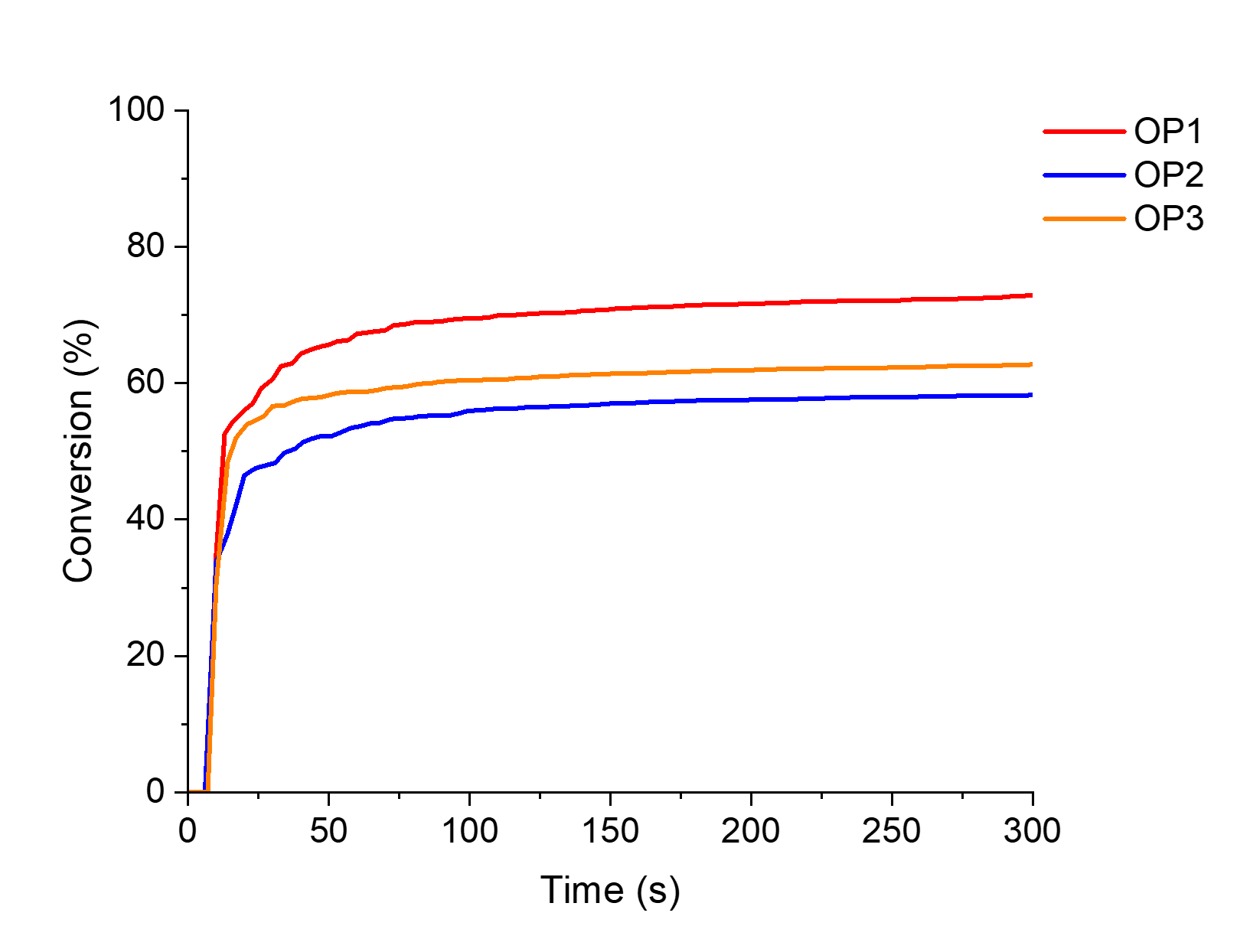


**Figure S3.** Photopolymerization kinetics of acrylate functions in TMPTA with photoinitiators OPIs (2×10^-5^ mol·g^-1^ TMPTA) in laminate (thickness ∼ 28 μm) irradiated by LED@405 nm. Exposure starts at t = 10 s.

**
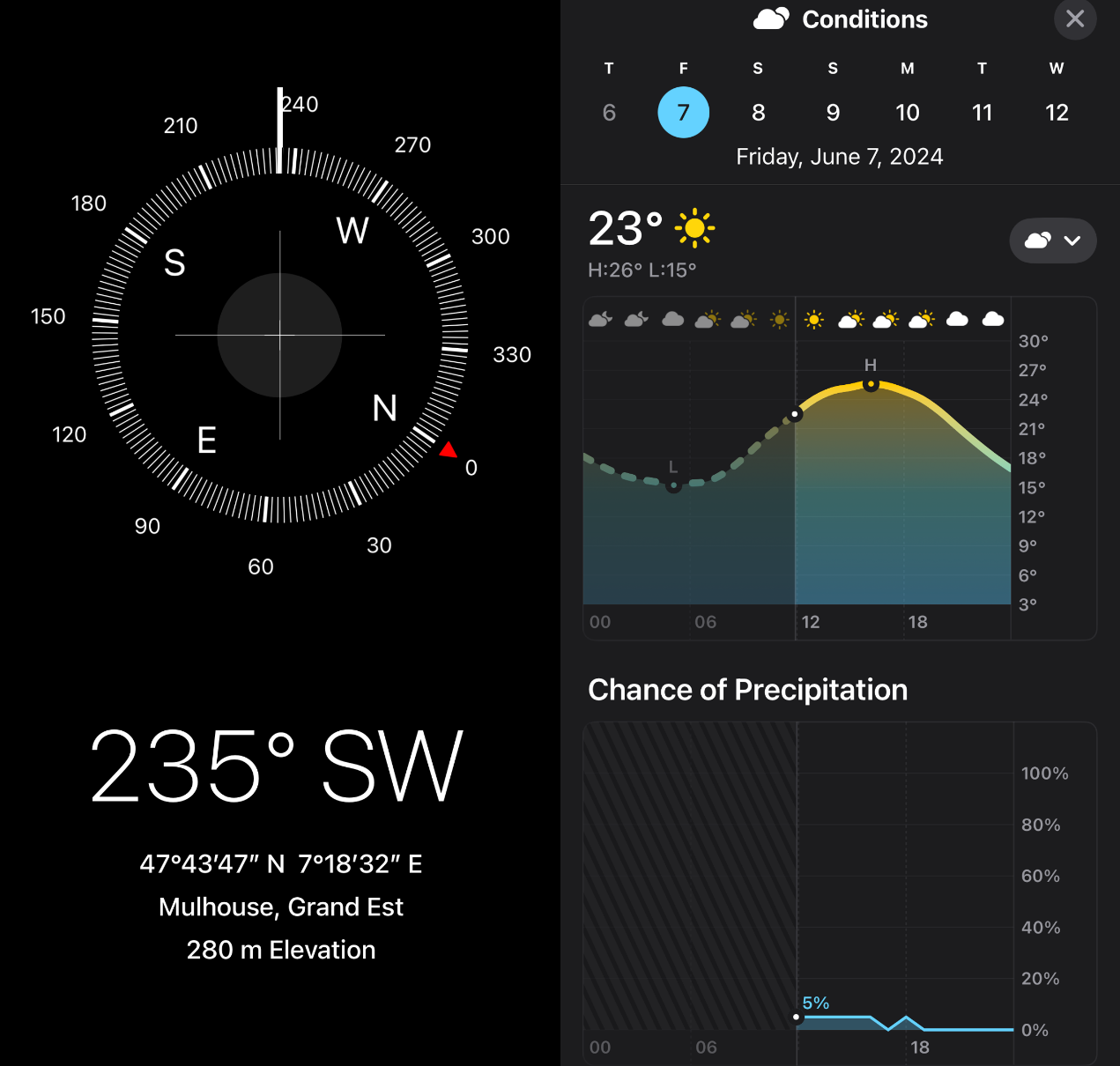
**

**Figure S4.** Environmental conditions for photopolymerization kinetics of TMPTA and ETPTA with photoinitiators (2×10^-5^ mol·g^-1^ TMPTA and 2×10^-5^ mol·g^-1^ ETPTA) irradiated by sunlight.

**
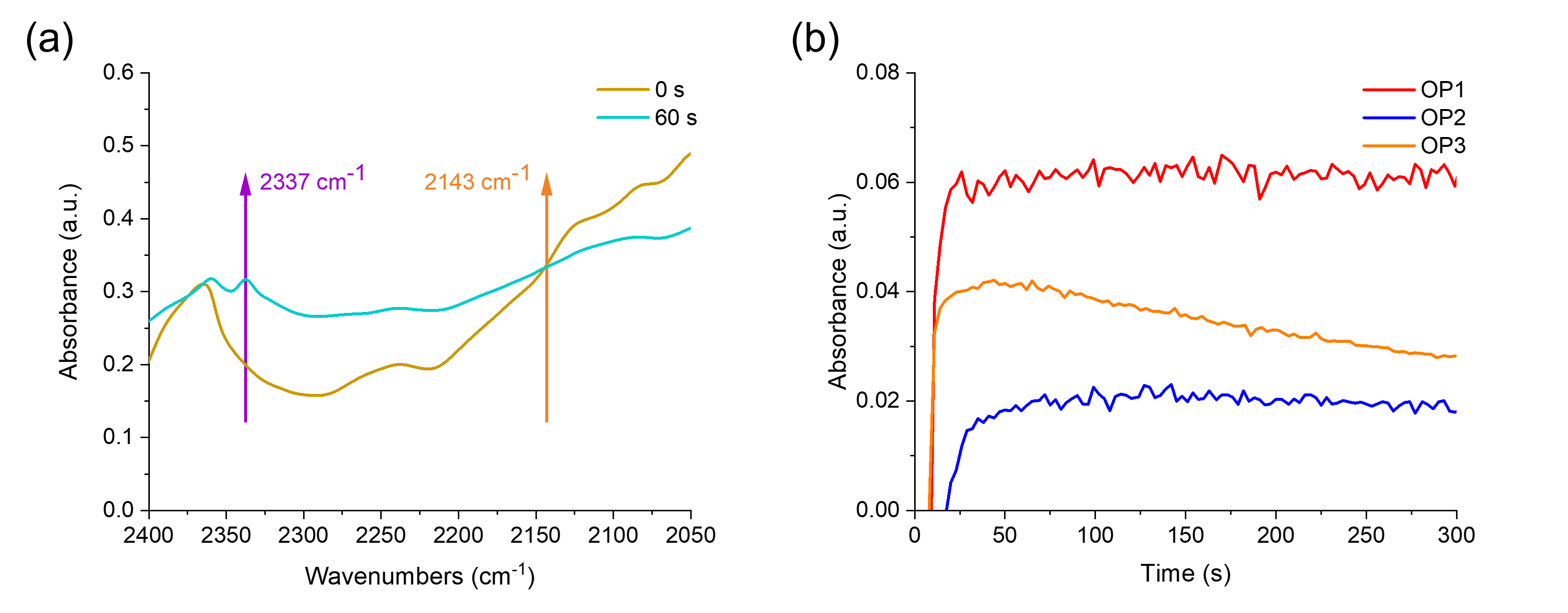
**

**Figure S5.** (a) Infrared spectrum of OP2 in TMPTA (2×10^-5^ mol·g^-1^ TMPTA) at t = 0 s and 60 s. (b) The curves of absorption intensity (absorbance vs irradiation time) of CO_2_ derived from OPIs/TMPTA.

**
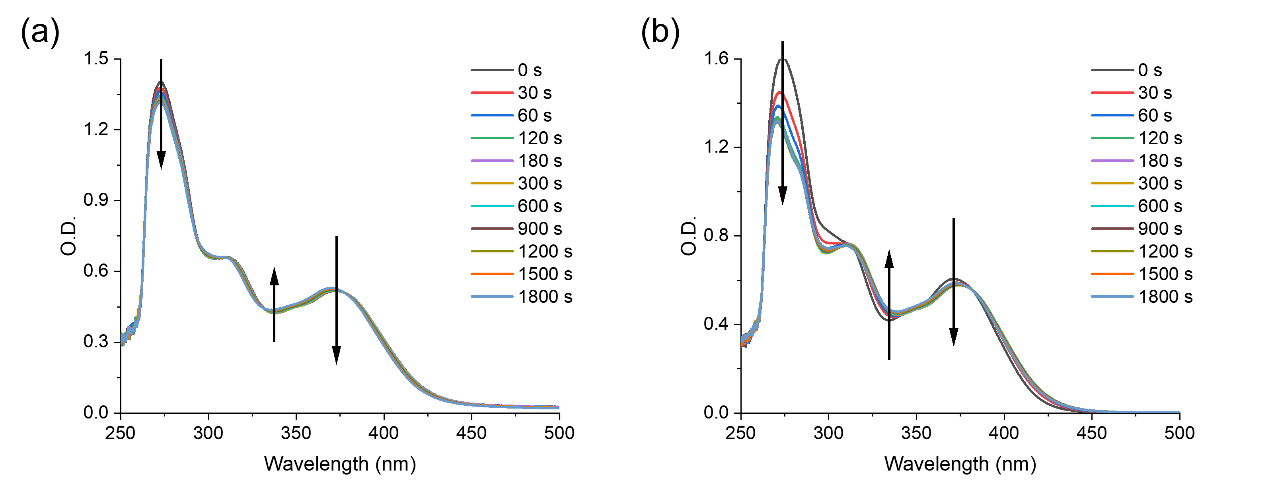
**

**Figure S6.** Steady state photolysis of (a) OP2 and (b) OP3 in acetonitrile exposed to LED@405 nm (concentration = 5 × 10^-5^ M).

**
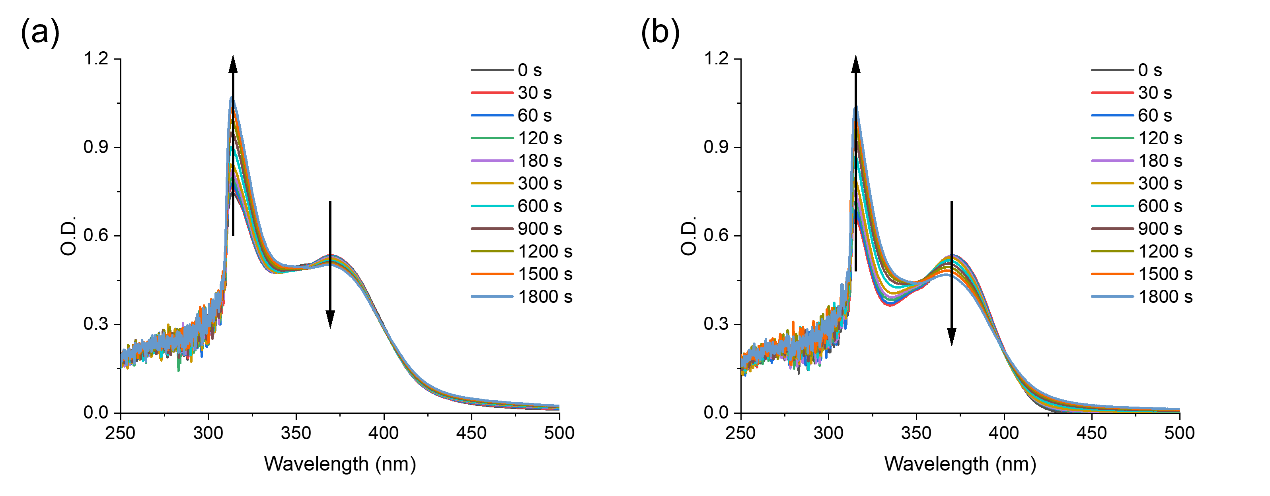
**

**Figure S7.** Steady state photolysis of (a) OP2 and (b) OP3 in TMPTA exposed to LED@405 nm (concentration = 5 × 10^-5^ M).


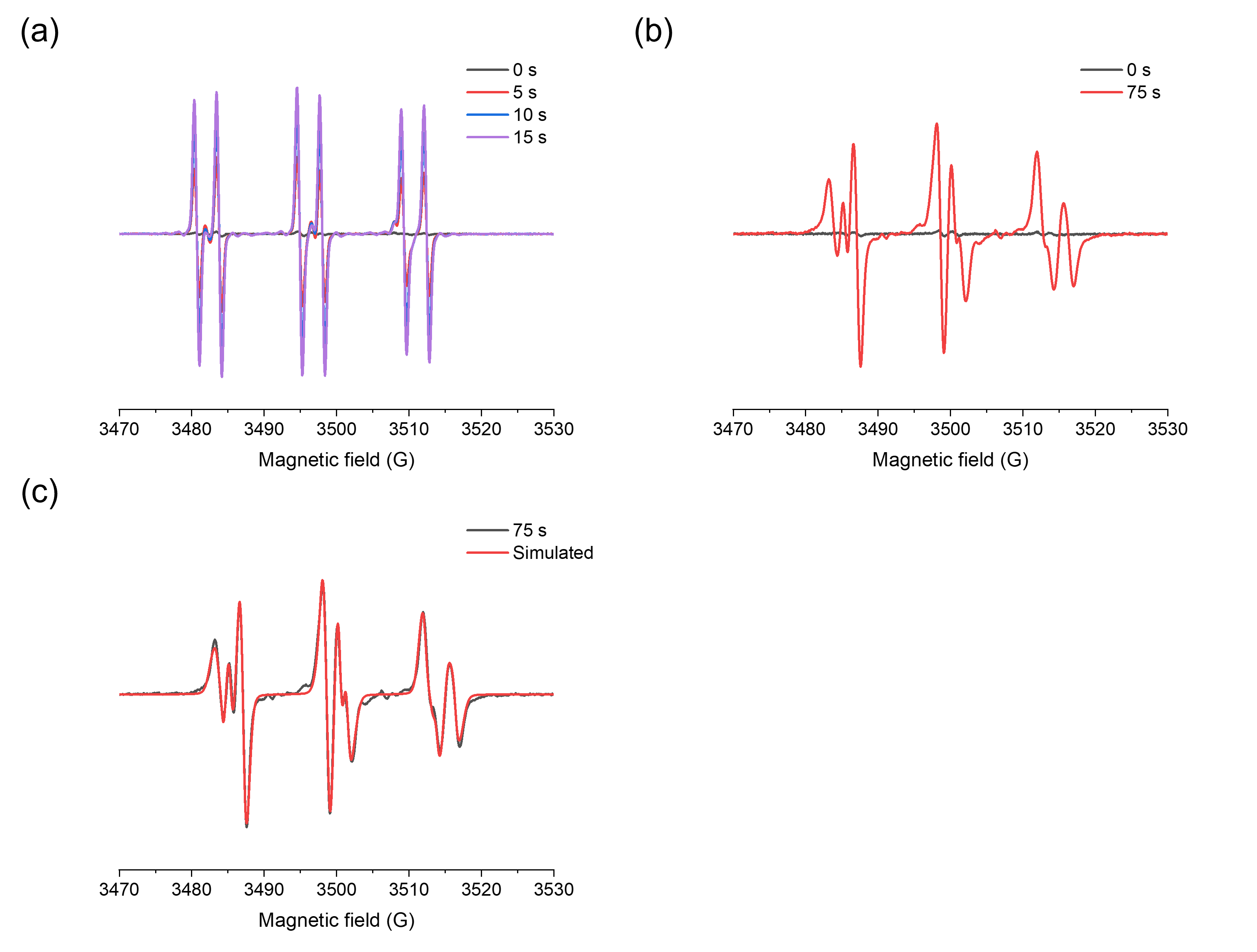


**Figure S8.** ESR spectra of (a) OP1 radical adducts exposed to LED@405 nm captured by PBN in *tert*-butylbenzene under N_2_ atmosphere for different exposure durations. ESR spectra of (b) OP2 radical adducts exposed to LED@405 nm captured by PBN in *tert*-butylbenzene under N_2_ atmosphere before irradiation (black) and after 75 seconds (red) and (c) superimposition of experimental and simulated ESR spectra of the methyl radical and PBN.


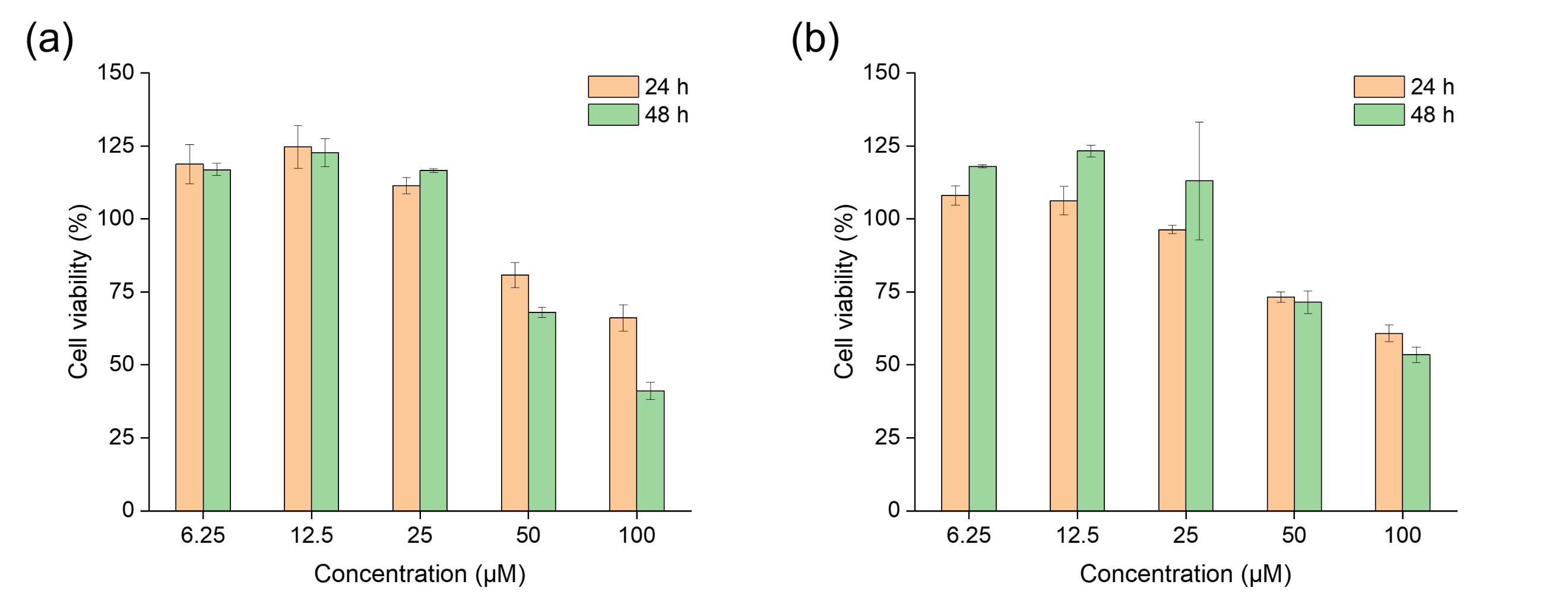


**Figure S9.** Cytotoxicity of different concentrations of (a) no-light TPO treated and (b) no-light OP1 treated groups in HUVECs after 24 h and 48 h of incubation.

3. Supplementary Tables

**Table S1.** Fluorescence lifetime of OPIs in acetonitrile (concentration = 5 × 10^-5^ M).

| OPIs | OP1 | OP2 | OP3 |
| --- | --- | --- | --- |
| Lifetime  (ns) | < 1.4 | < 1.4 | < 1.4 |

**Table S2.** Solubility of OPIs in TMPTA and ETPTA.

| OPIs | 2×10^-5^ mol·g^-1^ TMPTA | 1×10^-5^ mol·g^-1^ TMPTA | 2×10^-5^ mol·g^-1^ ETPTA | 1×10^-6^ mol·g^-1^ ETPTA |
| --- | --- | --- | --- | --- |
| OP1 | + | + | + | + |
| OP2 | + | + | + | + |
| OP3 | - | + | - | + |

Note: “+” indicates that OPIs were completely dissolved; “-” indicates that OPIs were not completely dissolved.

4. General information

All reagents and solvents were purchased from Aldrich or Alfa Aesar and used as received without further purification. Mass spectroscopy was performed by the Spectropole of Aix-Marseille University. ESI mass spectral analyses were recorded with a 3200 QTRAP (Applied Biosystems SCIEX) mass spectrometer. The HRMS mass spectral analysis was performed with a QStar Elite (Applied Biosystems SCIEX) mass spectrometer. Elemental analyses were recorded with a Thermo Finnigan EA 1112 elemental analysis apparatus driven by the Eager 300 software. ^1^H and ^13^C NMR spectra were determined at room temperature in 5 mm o.d. tubes on a Bruker Avance 400 or a Bruker Avance 300 spectrometer of the Spectropole: ^1^H (400 MHz), ^1^H (300 MHz), ^13^C (100 MHz), and ^13^C (75 MHz). All ^1^H chemical shifts were referenced to the solvent peak CDCl_3_ (7.26 ppm), DMSO-d_6_ (2.49 ppm) and the ^13^C chemical shifts were referenced to the solvent peak CDCl_3_ (77.0 ppm). The OP3 compound was synthesized according to published procedures.^1^

Synthesis of methyl-2-(((1-(9-dodecyl-6-nitro-9*H*-carbazol-3-yl)ethylidene)amino)oxy)-2-oxoacetate (OP1)

1-(9-Dodecyl-6-nitro-9*H*-carbazol-3-yl)ethan-1-one oxime (4.57 mmol) was added in anhydrous dichloromethane (DCM). Triethylamine (Et_3_N) (3 mL) was added to obtain a clear solution. Methyl chlorooxoacetate (5.5 mmol) was added. The flask was stirred at room temperature overnight. The crude reaction mixture was hydrolyzed with 20 mL of water then extracted with DCM (3×20 mL). The organic layer was then dried over MgSO_4_, and the solvent was removed under vacuum. The product was used without further purification (58 % yield).

^1^H NMR (400 MHz, CDCl_3_) δ 9.11 – 9.03 (m, 1H), 8.52 (d, *J* = 1.8 Hz, 1H), 8.43 – 8.41 (m, 1H), 8.07 (dd, *J* = 8.8, 1.8 Hz, 1H), 7.52–7.44 (m, 2H), 4.37 (t, *J* = 7.3 Hz, 2H), 4.00 (s, 3H), 2.63 (s, 3H), 1.90 – 1.86 (m, 2H), 1.34 – 1.23 (s, 18H), 0.87 (t, *J* = 6.7 Hz, 3H).

^13^C NMR (126 MHz, CDCl_3_) δ 165.68, 157.99, 155.86, 144.07, 143.36, 141.27, 126.46, 122.93, 122.59, 122.23, 120.58, 117.53, 110.08, 108.96, 53.83, 43.97, 32.00, 29.67, 29.61, 29.53, 29.41, 29.40, 29.03, 27.29, 22.78, 15.12, 14.21.

HRMS (TOF MS ES+) : m/z theor : 524.2755, found : 524.2764, [M+H]^+^ detected.

^1^H NMR spectrum of methyl-2-(((1-(9-dodecyl-6-nitro-9*H*-carbazol-3-yl)ethylidene)amino)oxy)-2-oxoacetate (OP1)


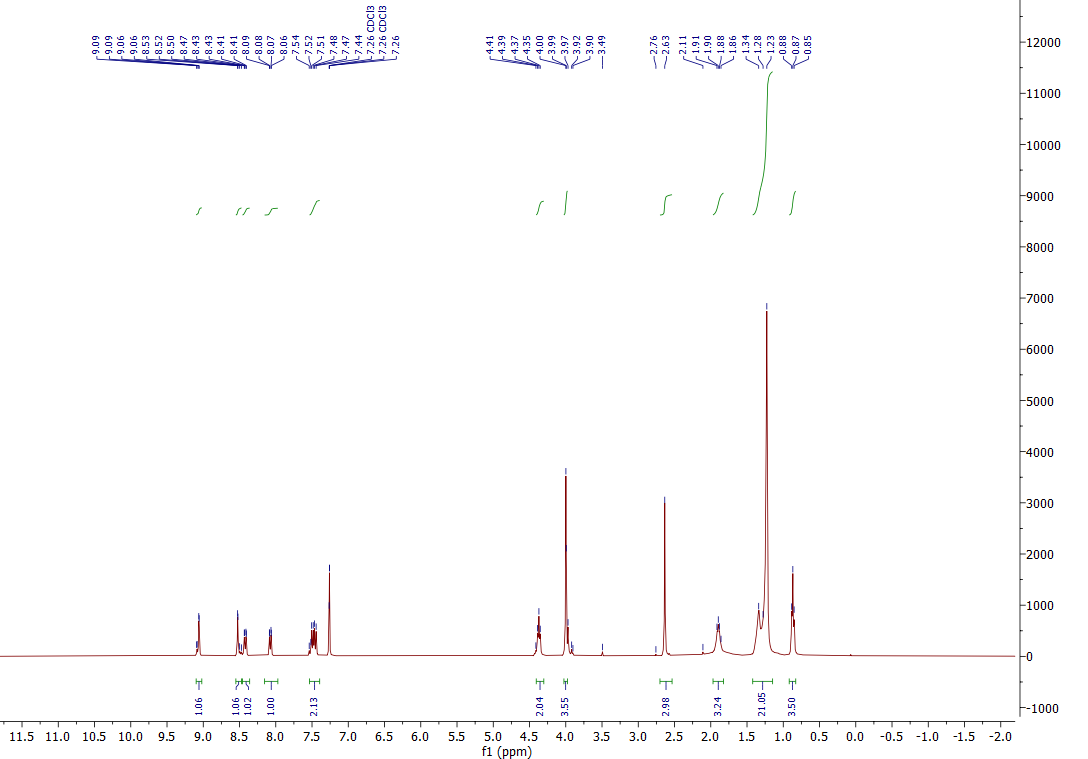


^13^C NMR spectrum of methyl-2-(((1-(9-dodecyl-6-nitro-9*H*-carbazol-3-yl)ethylidene)amino)oxy)-2-oxoacetate (OP1)


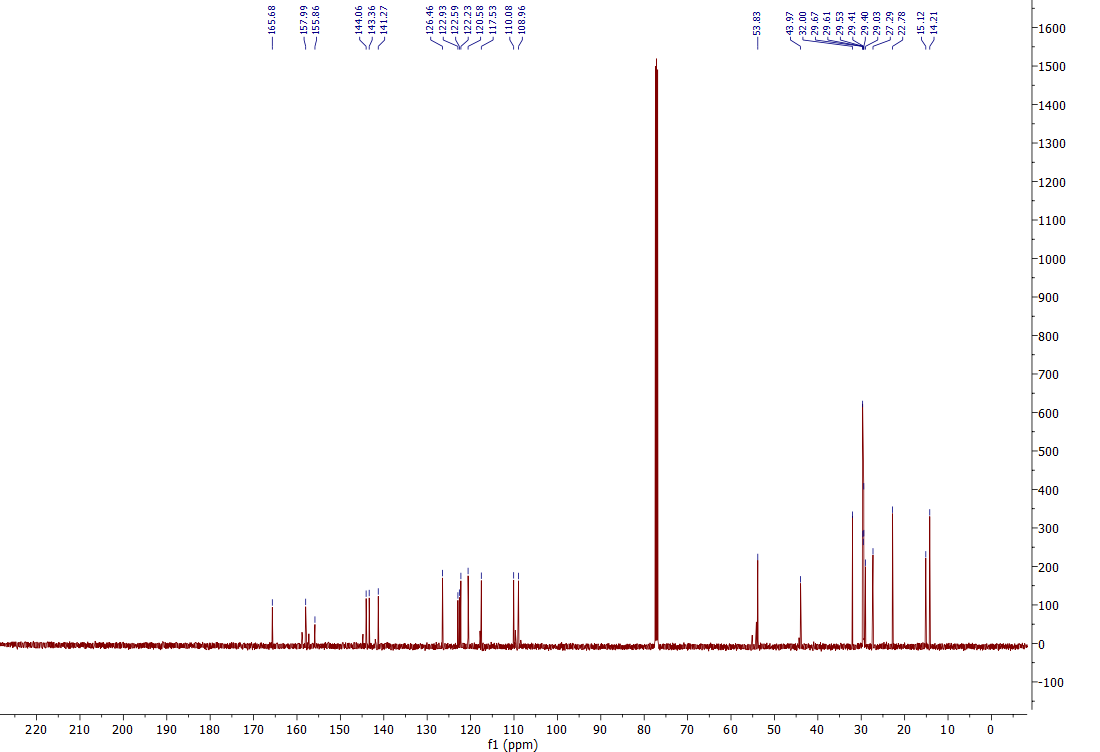


Synthesis of 1-(((1-(9-dodecyl-6-nitro-9*H*-carbazol-3-yl)ethylidene)amino)oxy)propane-1,2-dione (OP2)

To a solution of 1-(9-Dodecyl-6-nitro-9*H*-carbazol-3-yl)ethan-1-one oxime (1 mmol) in anhydrous DCM (25 mL), pyruvic acid (2 mmol), 1-ethyl-3-(3-dimethylaminopropyl)carbodiimide (EDCI) (2.5 mmol) and 4-dimethylaminopyridine (DMAP) (0.2 mmol) were added at room temperature. The reaction flask was sealed with a rubber septum and purged with argon in order to maintain an inert atmospher. The reaction mixture was stirred for 24 h at room temperature then quenched with water (10 mL). The reaction mixture was transferred to a separatory funnel and washed successively with 1M HCl (2×60 mL) and water. The organic layer was then dried over MgSO_4_, filtered and the solvent was removed under vacuum. The product was used without further purification (85% yield).

^1^H NMR (600 MHz, CDCl_3_) δ 9.01 – 8.96 (m, 1H), 8.48 – 8.42 (m, 1H), 8.37 (dd, *J* = 2.17, 9.00 Hz, 1H), 8.03 (dd, *J* = 1.61, 8.66 Hz, 1H), 7.48–7.40 (m, 2H), 4.34 (t, *J* = 7.28 Hz, 2H), 2.61 (s, 3H), 2.60 (s, 3H), 1.90 – 1.85 (m, 2H), 1.39 – 1.21 (m, 18H), 0.87 (t, *J* = 6.94 Hz, 3H).

^13^C NMR (126 MHz, CDCl_3_) δ 191.58, 165.54, 159.22, 144.76, 144.04, 143.30, 141.97, 141.26, 126.62, 126.41, 122.91, 122.58, 122.19, 120.48, 117.49, 110.02, 109.66, 108.92, 44.30, 43.94, 31.99, 29.67, 29.61, 29.53, 29.41, 29.39, 27.28, 27.24, 22.77, 14.99, 14.20.

HRMS (TOF MS ES+) : m/z theor : 530.2625, found : 530.2634, [M+Na]^+^ detected.

^1^H NMR spectrum of 1-(((1-(9-dodecyl-6-nitro-9*H*-carbazol-3-yl)ethylidene)amino)oxy)propane-1,2-dione (OP2)


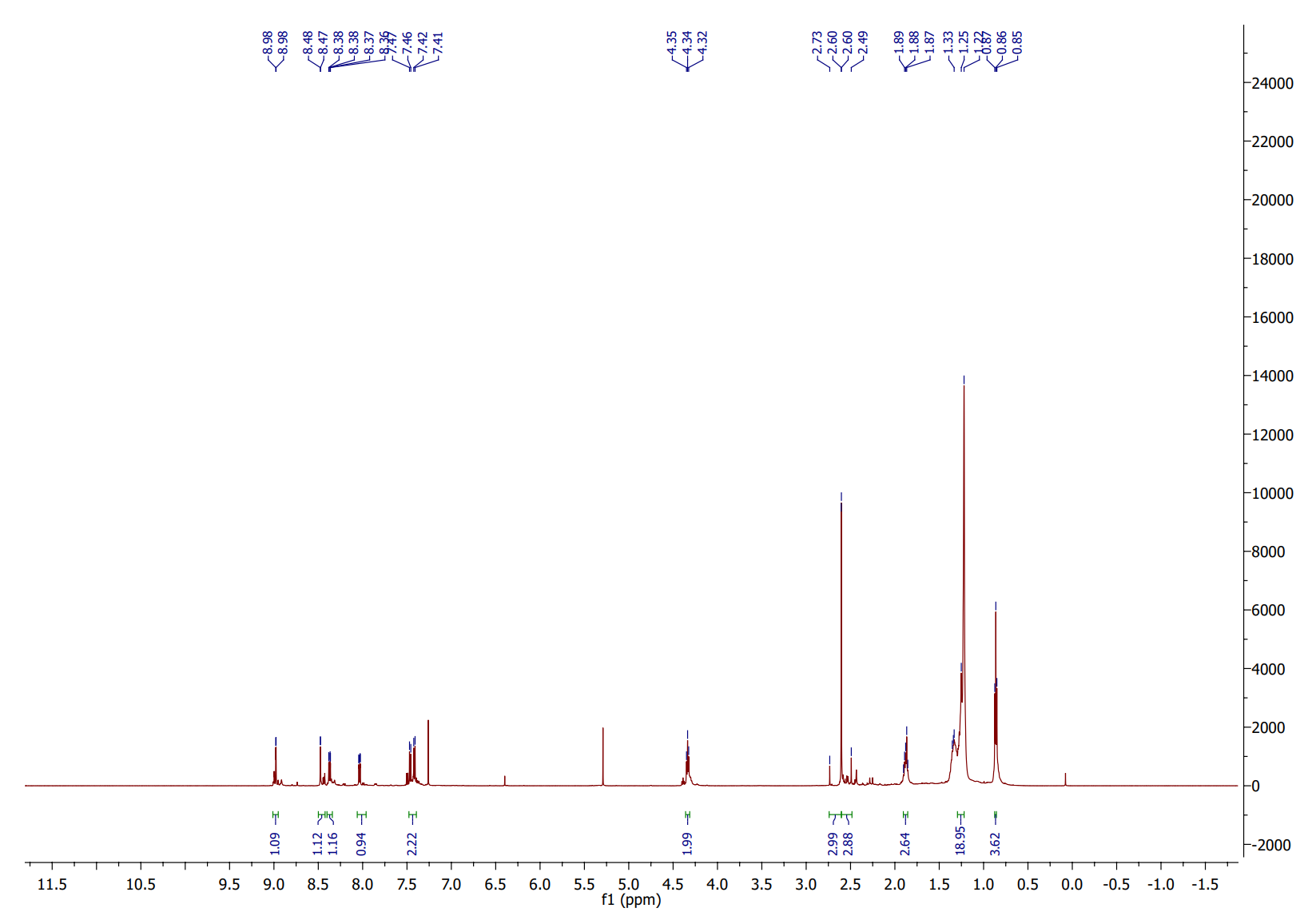


^13^C NMR spectrum of 1-(((1-(9-dodecyl-6-nitro-9*H*-carbazol-3-yl)ethylidene)amino)oxy)propane-1,2-dione (OP2)


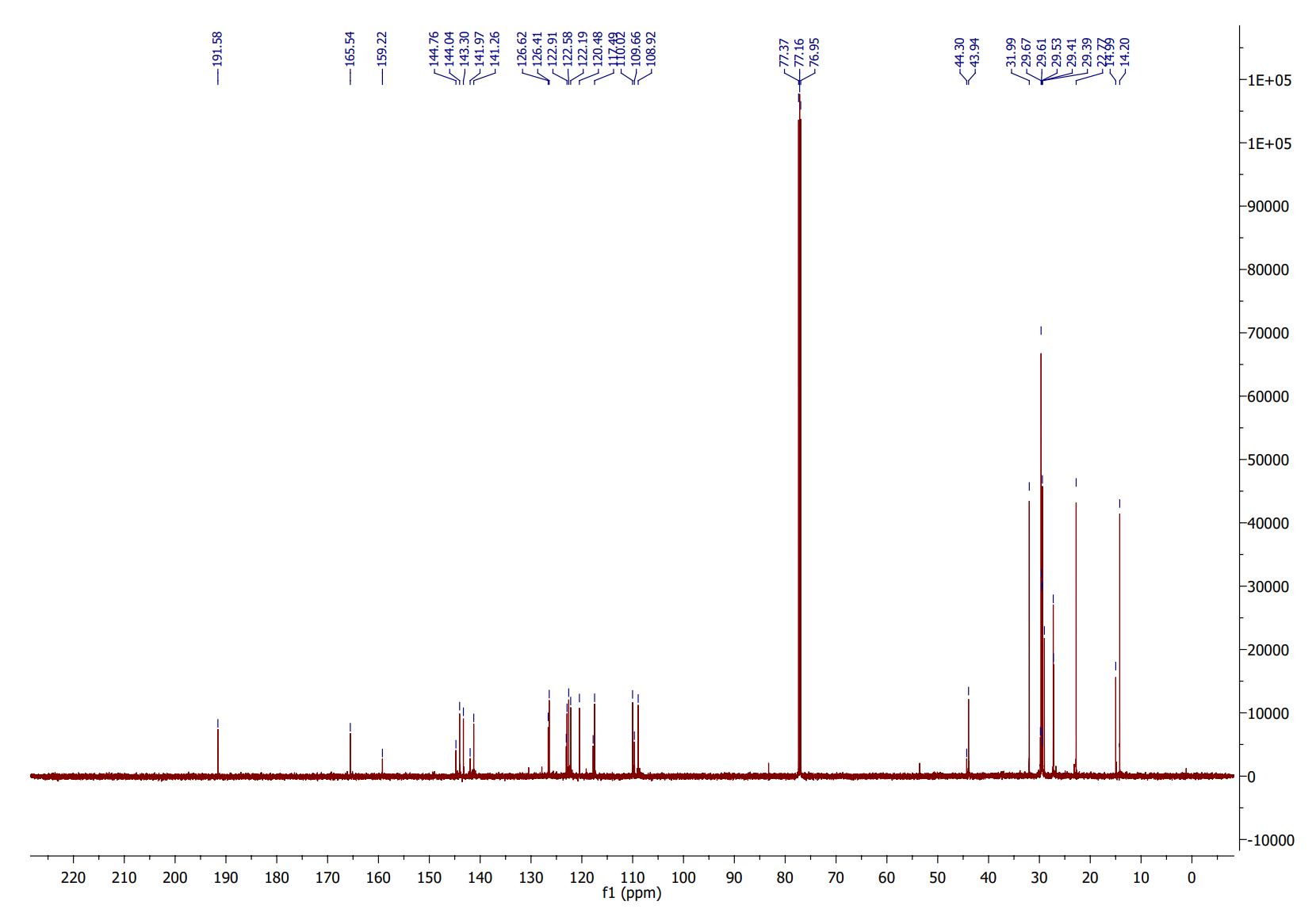


5. Reference

1. S. H. Liu, N. Giacoletto, M. Schmitt, M. Nechab, B. Graff, F. Morlet-Savary, P. Xiao, F. Dumur and J. Lalevée, *Effect of Decarboxylation on the Photoinitiation Behavior of Nitrocarbazole-Based Oxime Esters*, *Macromolecules*, 2022, **55**, 2475-2485.
